# Contrasting Responses of Protistan Plant Parasites and Phagotrophs to Ecosystems, Land Management and Soil Properties

**DOI:** 10.3389/fmicb.2020.01823

**Published:** 2020-08-05

**Authors:** Anna Maria Fiore-Donno, Tim Richter-Heitmann, Michael Bonkowski

**Affiliations:** ^1^Terrestrial Ecology Group, Institute of Zoology, University of Cologne, Cologne, Germany; ^2^Cluster of Excellence on Plant Sciences (CEPLAS), Cologne, Germany; ^3^Microbial Ecophysiology Group, Faculty of Biology/Chemistry, University of Bremen, Bremen, Germany

**Keywords:** biogeography, functional traits, soil protists, trophic guilds, Rhizaria, protistan plant pathogens, Cercozoa, Endomyxa

## Abstract

Functional traits are increasingly used in ecology to link the structure of microbial communities to ecosystem processes. We investigated two important protistan lineages, Cercozoa and Endomyxa (Rhizaria) in soil using Illumina sequencing and analyzed their diversity and functional traits along with their responses to environmental factors in grassland and forest across Germany. From 600 soil samples, we obtained 2,101 Operational Taxonomic Units representing ∼18 million Illumina reads (region V4, 18S rRNA gene). All major taxonomic and functional groups were present, dominated by small bacterivorous flagellates (Glissomonadida). Endomyxan plant parasites were absent from forests. In grassland, Cercozoa and Endomyxa were promoted by more intensive land use management. Grassland and forest strikingly differed in community composition. Relative abundances of bacterivores and eukaryvores were inversely influenced by environmental factors. These patterns provide new insights into the functional organization of soil biota and indications for a more sustainable land-use management.

## Introduction

A major aim in terrestrial ecology is to understand the drivers affecting the composition and functioning of the soil food web, and how its components contribute to ecosystem functions and services. Because soil biota are incredibly diverse, they are commonly aggregated into feeding groups (guilds or trophic species) to facilitate the understanding of their complex interactions (Scheu, 2002). Protists make up a substantial fraction (30–40%) of the soil eukaryotes (Shen et al., 2014; Lanzén et al., 2016; Ferreira de Araujo et al., 2018), and have long been recognized as a pivotal component of soil food webs (Hunt et al., 1987). Despite this, they have only recently been included in general models of ecosystem services (de Vries et al., 2014), probably because of their immense diversity. However, uncertainties in consumers’ preferences in soil food webs can strongly influence model predictions (Reyns et al., 2019). A main obstacle in food web models is the traditional, simplistic, but erroneous assumption that soil protists act only as bacterivores – however, protists occupy all trophic levels, also including, autotrophs, mixotrophs, saprotrophs, eukaryvores, omnivores, as well as parasites of animals and plants and their hyperparasites (Geisen et al., 2016; Bonkowski et al., 2019). This diversity of trophic roles of protists fundamentally affects the efficiency of the energy flow across the soil food web, which does not follow a single-track channel from small to large consumers as traditionally assumed (Potatov et al., 2019). Disentangling the multiple roles of protists will thus increase the trophic resolution of the soil food web and will have a major influence on network properties, such as topology and connectivity (Henriksen et al., 2019). Therefore, it is essential, not only to conduct large-scale molecular environmental sampling studies in soil, but also to attribute ecologically meaningful traits to the identified sequences. This is now facilitated by publicly available databases of protistan functional traits, including trophic guilds (Bjorbækmo et al., 2019; Dumack et al., 2019), and by community efforts to improve the reference databases for taxonomic annotation (del Campo et al., 2018).

Recent large-scale environmental sampling investigations of soil protists in natural and semi-natural environments gave insights into their multiple feeding modes, and how each trophic guild reacts to environmental filters and human-induced disturbances. Nonetheless, it must be noted that although the relative proportion of the main trophic guilds is known for planktonic protists (43% symbionts, 43% predators) (Bjorbækmo et al., 2019), such a comprehensive catalog has not yet been realized for soil. Only inventories limited, in space, by their specific aims, or by methodological biases- gave partially overlapping results: a worldwide soil survey indicated that protistan communities were largely composed of “consumers” with a minority of parasites and phototrophs (Oliverio et al., 2020). More precisely, in a single grassland soil targeting specific lineages, “consumers” were composed of bacterivores (67%) while omnivores (feeding on bacteria and other eukaryotes) and eukaryvores composed a noteworthy 25% (Fiore-Donno et al., 2019). Even less is known about the distribution of protistan plant pathogens in natural habitats: most of the protists that are known to interact with plants belong to the Stramenopiles-Alveolata-Rhizaria super group (Harosa or “SAR”), particularly those belonging to oomycetes (Stramenopiles) and Cercozoa (Rhizaria) (Hassani et al., 2018). For example, Schulz et al. (2019), investigating a gradient of land use intensification from tropical rain forest to plantations, reported significant shifts in the composition of protistan phagotrophs, phototrophs and plant pathogens. A study under controlled greenhouse conditions showed that organic fertilizer amendments reduced the relative abundance of plant pathogenic protists and increased that of bacterivores and omnivores (Xiong et al., 2018). However, in a field study, plant parasites were favored by organic fertilization (Harkes et al., 2019). Thus, currently a global and coherent picture of the responses of protistan plant pathogens to fertilization and other anthropogenic changes cannot be inferred.

Elucidating the diversity, dynamics and the environmental drivers of protistan plant pathogens is important for a sustainable land management, not only in agrosystems but also in natural ecosystems, because of their importance as drivers of patterns of plant diversity and productivity (Mitchell, 2003; Schnitzer et al., 2011). However, we are lacking baseline data on their occurrence in natural reservoirs, such as grasslands and forests (Stukenbrock and McDonald, 2008; van der Heijden et al., 2008). Cercozoa (Cavalier-Smith, 1998), a highly diverse phylum of c. 600 described species (Pawlowski et al., 2012), is a major protistan lineage in soil (Geisen et al., 2015; Grossmann et al., 2016; Lanzén et al., 2016; Ferreira de Araujo et al., 2018), comprising a vast array of functional traits in morphologies, nutrition and locomotive modes (Burki and Keeling, 2014). Recently the phylum Endomyxa was separated from Cercozoa (Bass et al., 2018; Cavalier-Smith et al., 2018), and it is of particular interest for containing *inter alia* plant pathogens of functional and economic significance, including important transmitters of plant viruses and agents of root tumors (e.g., club root disease) (Neuhauser et al., 2014; Bass et al., 2019).

How land use intensification and human disturbances influence the biogeographies of soil microbes has recently been given much attention. To quantify land use intensity (LUI) in grasslands, an index, integrating quantitative data on mowing, grazing and fertilization (Blüthgen et al., 2012), has been widely applied in the framework of the German Biodiversity Exploratories. Increasing land use intensity had strong overall effects on ecosystem multifunctionality (Allan et al., 2015) and imperiled plant and animal community stability (Blüthgen et al., 2016). In particular, it was shown that plant species richness decreased along increasing land use gradients (Egorov et al., 2014). With regard to soil microorganisms, the LUI index only weakly influenced bacterial communities (Kaiser et al., 2016), slightly increased ammonia oxidizing prokaryotes (Keil et al., 2011) and had no effect on protistan community assemblages as detected by T-RFLP (Glaser et al., 2015). However, it was demonstrated that specific environmental responses of protists remained undetectable when general eukaryotic PCR primers were used, but instead could be observed by the more thorough coverage of single lineages through taxon-specific primers (Lentendu et al., 2014).

Our aims were thus: (i) the assessment of the diversity of Cercozoa and Endomyxa, classified into trophic guilds, in grassland and forest along regional and land use intensification gradients; (ii) the identification of environmental factors driving the distribution of the cercozoan and endomyxan communities, with a special focus on plant pathogens. Our study took place in 150 grassland and 150 forest sites of the German Biodiversity Exploratories, sampled in 2011 and 2017 (600 soil samples in total). We tested the hypothesis that land use intensification would lead to shifts in the relative abundance of trophic guilds, increasing plant pathogens. Furthermore, we investigated to which degree grasslands and forests constituted natural reservoirs of the endomyxan plant parasites. Elucidating these patterns, both at regional and landscape scale, will provide new insights into the functional organization of the soil ecosystem and may give indications for a more sustainable land-use management.

## Materials and Methods

### Study Sites, Soil Sampling and DNA Extraction

Our study took place in three German Biodiversity Exploratories, i.e., the Biosphere Reserve Schorfheide-Chorin in the State of Brandenburg, the National Park Hainich and its surroundings in the State of Thuringia and the Biosphere Reserve Schwäbische Alb in the State of Baden-Württemberg (Fischer et al., 2010). Each exploratory comprises 50 grassland sites from extensive pastures to highly fertilized meadows and 50 differently managed forest sites. Each site contains a study plot of 20 × 20 m. From all study plots, 300 soil samples were collected in a coordinated joint sampling campaign within 14 days in April 2011 and a second one in April 2017. From each plot 14 soil cores of 5 cm diameter were taken every 3 m along two transects of 20 m each in grassland and 40 m each in forest, oriented North-South and East-West, employing a soil corer. The surface layer (0–10 cm) was collected, after removing plants, pebbles and conspicuous roots. For determining edaphic properties, soil cores (1.2–4.7 kg) from each plot were sieved (2 mm mesh size), mixed, homogenized and kept at 4 °C for further analyses. Detailed sampling procedures, methods to measure edaphic properties and regional characteristics were described elsewhere (Birkhofer et al., 2012; Solly et al., 2014). Environmental parameters for all grassland (Supplementary Table S1) and forest sites (Supplementary Table S2) are given. Mean values are compared between regions and year of sampling (Supplementary Table S3). Soil DNA was extracted in 2011 and in 2018, from 400 mg of soil (not sieved), 3- to 6-times, using the DNeasy PowerSoil Kit (Qiagen GmbH, Hilden, Germany) following the manufacturer’s protocol, to obtain a sufficient amount to be shared between research groups of the Biodiversity Exploratories.

### Primer and Barcode Design, Amplification and Sequencing

We modified the primers specific for Cercozoa from Fiore-Donno et al. (2018) to better match the parasitic lineages Phytomyxea and Proteomyxidea in Endomyxa. The first PCR was conducted with a mixture of the forward primers S615F_Cerco + S615F_Phyt, 50% each (all primers 5′-3′) GTTAAAAAGCTCGTAGTTG and GTTAAAARGCTCGTAGTCG and reverse S963R_Phyt CAACTTTCGTTCTTGATYAAA; the second PCR was conducted with barcoded primers (Supplementary Table S4), forward S615F_Cer GTTAAAARGCTCGTAGTYG and reverse S947R_Cer AAGARGAYATCCTTGGTG. The barcodes consisted in eight-nucleotide-long sequences appended to the 5′-ends of both the forward and the reverse primers, because tagging only one primer leads to extensive mistagging (Esling et al., 2015). To design the barcodes, we first used barcrawl (Frank, 2009) to obtain a list of barcodes with a balanced nucleotide content (no homopolymers), not folding on themselves nor to themselves and the attached primer (no “hairpin”), not forming heteroduplexes with the corresponding primer and having at least 3 bases differences between them. In addition, using custom R scripts, we selected from the previous list only the barcodes that did not match the consensus of the reference alignment flanking the primer region and without cross-dimerization between each combination of primer+barcodes. We designed 18 barcoded versions for the forward and the reverse primers (Supplementary Table S4), allowing for 324 possible combinations to label samples of which only 150 were used, since it is advisable to leave a proportion of unused combinations to decrease mistagging (Esling et al., 2015). Barcoded primers were specifically ordered for NGS application to Microsynth (Wolfurt, Austria).

For the amplification, we incorporated 1 μl of 1:10 soil DNA template for the first PCR round and 1 μl of the resulting amplicons as a template for a following semi-nested PCR. We employed the following final concentrations: GreenTaq polymerase (Fermentas, Canada) 0.01units, buffer 1×, dNTPs 0.2 mM and primers 1 μM. The thermal programme consisted of an initial denaturation step at 95°C for 2 min, 24 cycles at 95°C for 30 s, 50°C for 30 s, 72°C for 30 s; and a final elongation step at 72°C for 5 min. The number of PCR cycles was kept at 24 since chimera formation arises dramatically after 25 cycles (Michu et al., 2010). All PCRs were conducted twice to reduce the possible artificial dominance of few amplicons by PCR competition (2 × 10 μl for the first and 2 × 27 μl for the second PCR), and the two amplicons were pooled after the second PCR.

The amplicons were checked by electrophoresis and 25 μl of each were purified and normalized using SequalPrep Normalization Plate Kit (Invitrogen GmbH, Karlsruhe, Germany) to obtain a concentration of 1–2 ng/μl per sample, and the 150 samples were pooled. We conducted 4 Illumina runs, for each year (2011 and 2017) and for grassland or forest. During the library preparation, amplicons were end-repaired, small fragments were removed, 3′ ends were adenylated, and Illumina adapters and sequencing primers were ligated (TruSeqDNA PCR-Free, Illumina Inc., San Diego, CA, United States). The library was quantified by qPCR, performed following the manufacturer’s instructions (KAPA SYBR FAST qPCR Kit, Kapa Biosystems, Wilmington, MA, United States) on a CFX96 Real Time System (Bio-Rad, Hercules, CA, United States). Sequencing was performed with a MiSeq v3 Reagent kit of 300 cycles (on a MiSeq Desktop Sequencer (Illumina Inc., San Diego, CA, United States) at the University of Geneva (Switzerland), Department of Genetics and Evolution.

### Sequences Processing

Paired reads were assembled using MOTHUR v.39.5 (Schloss et al., 2009) (which was also used in the following steps) allowing no differences in the primers and the barcodes, no ambiguities and removing assembled sequences <300 bp and with an overlap <100 bp (Table 1). The quality check and removal/cutting of low-quality reads were conducted with the default parameters. Reads were sorted into samples via detection of the barcodes (Supplementary Table S4) and sequences renamed with the sample, year and “G” for grassland or “F” for forest. After clustering identical sequences together, all reads were assembled in one file. Sequences were clustered into Operational Taxonomic Units (OTUs) using VSEARCH (Rognes et al., 2016) with abundance-based greedy clustering (agc) and a similarity threshold of 97%. Clusters represented by less than 0.01% of the reads were removed as they likely represented amplification or sequencing noise (Fiore-Donno et al., 2018). Using BLAST+ (Camacho et al., 2008) with an *e*-value of 1^*e*–50^ and keeping only the best hit, sequences were identified in the PR2 database (Guillou et al., 2013) and non cercozoan sequences were removed. Sequences were aligned with the provided template (Fiore-Donno et al., 2018) with a penalty for opening gaps of −5. Chimeras were identified using UCHIME (Edgar et al., 2011) as implemented in mothur and chimeric sequences were removed (Table 1). The results are shown as a table with the OTUs abundance/site, their taxonomic assignment according to the best hit by Blast and their functional assignment according to Dumack et al. (2019) (Supplementary Table S5). The relative abundance of each cercozoan taxonomic level was illustrated using Sankey diagram generator V1.2^1^ and refined with the open-source vector graphic editor Inkscape^2^.

**TABLE 1:** Quality estimates and error rate for each run; initial, assembled, quality-trimmed, and unique number of reads for each run. Number of reads retrieved at each step of the sequence processing for the 4 assembled runs.

Illlumina runs	>Q30 %	% Error rate	Reads	Assembled reads	% Assembled reads	Quality trimmed	% Unique reads	4 runs assembled, total reads	Clusters at 97% sim. (=OTUs)	OTUs - rare (<0.01%)	Representing sequences	OTUs genuine	OTUs aligned	OTUs non-chimeric	Representing sequences
Grassland 2011	92	1.45	19′506′515	9′891′272	51	7′112′425	52	26′236′218	1′211′718	3′155	21′417′500	2′907	2′861	**2**′**101**	**17**′**679**′**414**
Grassland 2017	94	1.6	17′012′202	9′954′913	59	7′349′875	59								
Forest 2011	92	1.55	24′591′186	13′708′253	56	5′345′392	59								
Forest 2017	93	1.51	26′768′732	11′633′737	43	6′428′526	60								

### Statistical Analyses

All statistical analyses were carried out within the R environment (R v. 3.5.1) (R Development Core Team, 2014) on the OTU abundance/sites table (Supplementary Table S5) and the environmental parameters (Supplementary Tables S1, S2), the latter normalized by the K-nearest neighbors. Unless otherwise specified, community analyses were performed with the package vegan (Oksanen et al., 2013).

#### Alpha Diversity

To evaluate if more sampling and sequencing effort would have revealed more OTU richness, we carried out an analysis based on OTUs accumulation curves, function *specaccum*, method rarefaction and 1,000 random permutations; species richness was extrapolated using the function *specpool*. Alpha diversity estimates were based on relative abundances of OTUs (function *decostand*, method “total”); Shannon diversity and Pielou’s evenness were obtained with the function *diversity*.

#### Beta-Diversity

Variation partitioning (function *varpart* applied to the Hellinger-transformed OTUs dataset and using RDA, function *rda*) was applied to assess the amount of explained beta diversity by the factors region, year of collection (2011, 2017) and or ecosystem (grassland vs. forest). Beta diversity between regions and ecosystems was inferred by Principal Coordinate Analysis (PCoA, function *cmdscale*), using Bray-Curtis dissimilarities (function *vegdist*, method “bray”) on the relative abundances of OTUs, then plotted with the package ggplot2. A distance-based redundancy analysis (dbRDA) on Bray-Curtis dissimilarities (function *dbrda*) was used to investigate the effect of environmental factors on the beta-diversity of cercozoan and endomyxan communities. Parsimonious models were selected by the function *ordistep* with default parameters based on 999 permutations, and only significant results were shown in dbRDA. All available parameters were tested for co-correlation according to variance inflation factors (function *vif.cca*). Among the soil parameters measured in the framework of the Biodiversity Exploratories (Supplementary Tables S1, S2), soil carbon (total, organic, and inorganic) and nitrogen (total N) were all co-correlated; based on slightly higher significance in the models organic carbon was kept. Among the three co-correlated descriptors of soil texture, soil clay content was chosen over sand and silt contents.

Because the relative abundance is an important component of the beta diversity, the effects of environmental parameters on the relative abundances of the trophic guilds were tested with general linear models (function *glm*, core package). They were then subjected to the general linear hypothesis test (function *glht*, package multcomp) with Tukey’s test for multiple comparisons of means and a heteroskedasticity-consistent covariance matrix estimation (function *vcovHC*, package sandwich).

## Results

### Sequencing Results

We obtained c. 20 million reads per run (Table 1). Our primers were highly specific: the proportion of non-targeted OTUs only accounted for 8%. We obtained 2,101 genuine cercozoan OTUs from 600 grassland and forest soil samples representing nearly 18 million sequences. Nine samples were removed in 2011 due to insufficient yield (<10,000 reads/sample), probably due to problems during the amplification: SEG16, SEF31, SEF33, HEF07, HEF21, HEF23, HEF33; and in 2017: HEF07, SEF18. The remaining 591 samples yielded on average 29,817 reads/sample (min. 10,300, max 64,130, SD 9,784). The average number of OTUs per sample was 904 (maximum 1,365, minimum 254, SE 170.5). A database with the OTU abundance per sample, taxonomic assignment and estimated functional traits is provided (Supplementary Table S5). Our sequencing and sampling efforts were sufficient, since the actual OTU richness was reached after only c. 235,000 sequences (Supplementary Figure S2A) and at 65 samples (Supplementary Figure S2B).

### Taxonomic and Functional Diversity of Cercozoa and Endomyxa

**FIGURE 1:**
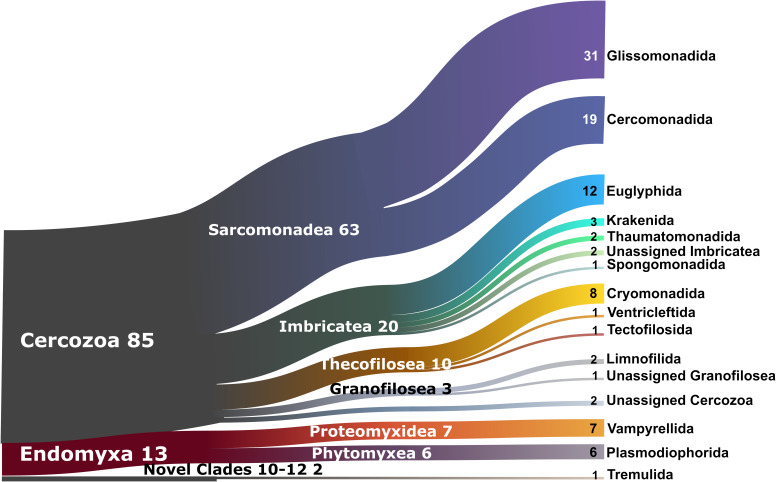
Sankey diagram showing the relative contribution of the cercozoan and endomyxan OTUs to the taxonomic diversity. Taxonomical assignment is based on the best hit by BLAST. From left to right, names refer to phylum (Cercozoa, Endomyxa), class (ending -ea) and orders (ending -ida). Numbers are percentages of OTUs abundance, taxa representing <1% are not shown.

**FIGURE 2:**
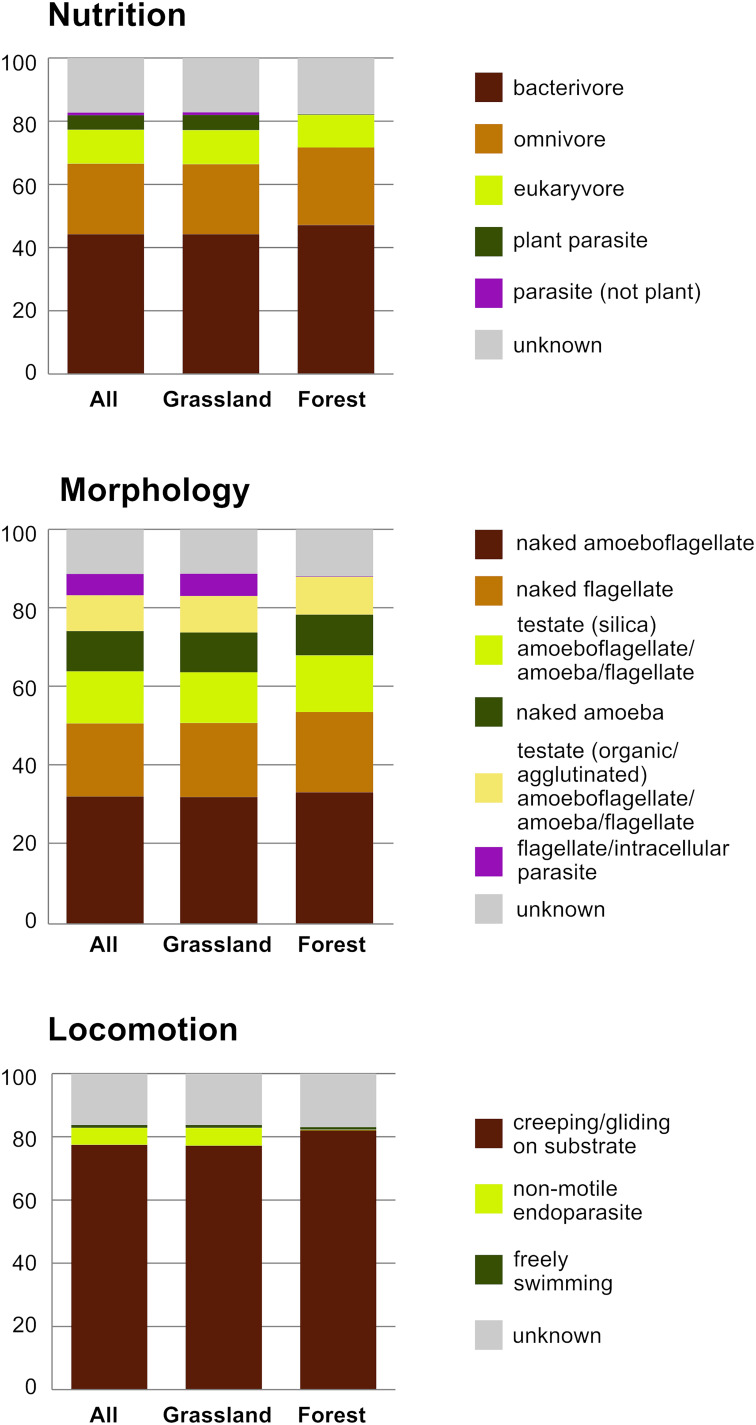
Histograms of the relative proportion of functional diversity classes of Cercozoa and Endomyxa (nutrition, morphology, and locomotion); All sites, and the two ecosystems separately.

The 2,101 OTUs represented 329 unique Blast best hits. Only 28% of the OTUs were 97–100% similar to any known sequence (Supplementary Figure S1). At a high taxonomic level, the majority of the OTUs could be assigned to the phylum Cercozoa (75%), the remaining to Endomyxa (25%) (Figure 1). Within Cercozoa, the class Sarcomonadea dominated (40%), composed of Glissomonadida and Cercomonadida. Glissomonadida is mostly composed of small flagellates while Cercomonadida mainly comprise amoeboflagellates. Other main lineages were represented by testate amoeba with silica shells in Euglyphida (13%, Imbricatea) and those with mainly organic tests in Cryomonadida (12%, Thecofilosea). Endomyxa was composed of the plant parasitic Plasmodiophorida (14%, Phytomyxea) and amoeboid Vampyrellida (11%, Proteomyxidea). Naked cells (flagellates, ∼19% and amoeboflagellates, ∼32%, amoebae, ∼10%) together constituted the predominant morphotypes, whereas testate cells (∼22%) were less frequent. Among the latter, cells with organic/agglutinated test accounted for ∼9% and those with a siliceous test for ∼13–14%. Considering trophic guilds, nearly 50% of the OTUs were bacterivores, but omnivores (∼23%) and eukaryvores (∼10%) also contributed sizeable proportions, in both grasslands and forests. Compared to these predominant functional groups, the plant parasites (∼5%) and hyperparasites of Oomycota (∼0.8%) made up a much smaller proportion in grasslands and surprisingly were absent from forest soils. Significant differences between the two ecosystems (Tukey’s HSD test, *p* < 0.01) were found for each nutritional guild except omnivores (“nutrition unknown” were not tested). The overwhelmingly dominant locomotive mode was creeping/gliding in forest and grassland soils, suggesting a predominance of biofilm feeders (Figure 2).

### Differences in Alpha and Beta Diversity Between Ecosystems

**FIGURE 3:**
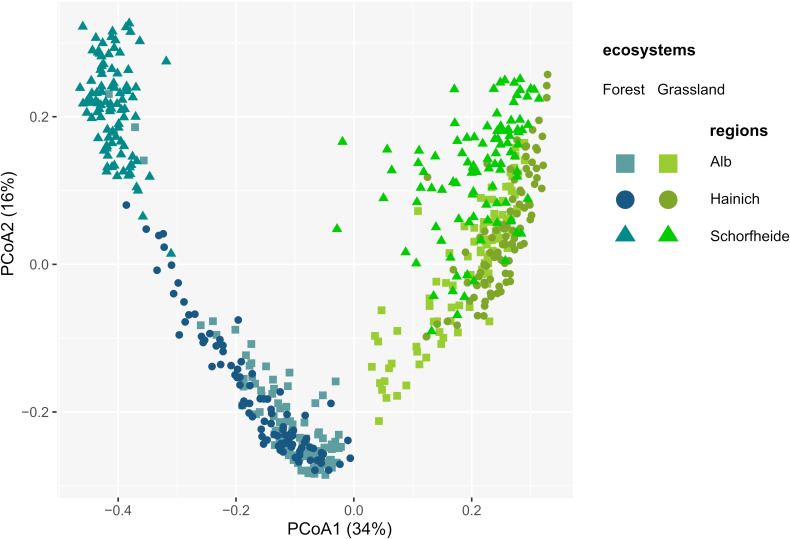
Principal Component Analysis of the Bray-Curtis dissimilarity indices between cercozoan and endomyxan OTUs, showing dramatically different communities between the two ecosystems and in a lesser extent between regions.

A total of 2,004 and 1,808 OTUs were retrieved in grassland and forest sites, respectively. 1,712 OTUs (81%) were shared between grasslands and forests, while only 293 and 97 OTUs were unique to grasslands and forests, respectively. Most OTUs were shared between regions, except two that were absent in Alb, one in Hainich, and three in Schorfheide (Supplementary Table S5), and one present only in Schorfheide. Consequently, differences in protistan alpha diversity depended more on relative abundances than on OTUs richness. Thus, higher alpha diversity of Cercozoa and Endomyxa in forests compared to grasslands were likely explained by a higher evenness (Supplementary Figure S3). Despite the almost ubiquitous presence of protistan OTUs in soil of grassland and forest, their communities dramatically differed between the two ecosystems. PCoA showed a strong arch effect indicating the overwhelming importance of the first component (*x*-axis), which separated forest and grassland sites, explaining 34% of the variance of Bray-Curtis distances (Figure 3). The second PCoA component explained 16% of variance and roughly reflected the regional differences along the south-north gradient, with the communities of Schorfheide standing out, especially in forests (Figure 3). This was confirmed by variance partitioning, which indicated that the ecosystem (forest vs. grassland), the region (Alb, Hainich, and Schorfheide) and the year of sampling (2011 vs. 2017) together accounted for 42.7% (adjusted R^2^) of the total variation in beta diversity. Ecosystem explained 30.4% of the variation, followed by differences between regions (9.7%) and year of sampling (2.4%). Differences in soil properties and management practices always explained more variance of beta diversity in forests (R^2^ values 10.2–53.2) than in grasslands (R^2^ values 3.6–7.3) (Supplementary Table S6). Most of the edaphic conditions significantly differed between regions: soils were more acidic in the grasslands of Hainich and in the forests of Schorfheide. The grasslands and forests of Schorfheide had the highest and lowest organic carbon content, respectively. The grassland and forest soils of the three regions differed significantly in their soil clay content, with a increase from Schorfheide to Alb. The LUI index did not differ between regions, but fertilization was significantly lower in Schorfheide (Supplementary Table S3).

### Major Drivers of Cercozoan and Endomyxan Communities Turnover Selected by Models

The most parsimonious models identified a strong influence of soil type and soil C/N ratio on protistan community composition for both grassland and forest (Supplementary Table S6), although edaphic factors had a stronger influence on protistan communities in forest than in grassland. Most important factors were pH, clay content, C/N ratio, soil type and the identity of the dominant tree species (Supplementary Table S6). This was true for the whole community of OTUs as well as for the individual trophic guilds. Bacterivores were further influenced by differences in the soil organic carbon content. At the regional level, organic C was a stronger determinant than soil type (Supplementary Table S6).

In grasslands, protistan communities and trophic guilds were influenced by indicators of human disturbance, in particular, differences in grassland management intensity (pastures, mowed meadows, meadows), and land use intensification (LUI index), such as grassland fertilization. At the regional level, differences in land use intensity had a significant influence on protistan communities in Schorfheide and Hainich grasslands, with the additional effect of mowing intensity in Hainich.

### Changes in Relative Abundances of Trophic Guilds to Environmental and Edaphic Parameters

Differences in the relative abundances of trophic guilds between regions were more pronounced in forest than in grassland (Supplementary Figures S4, S5). In grassland, the relative abundance of trophic guilds differed between regions, with bacterivores and omnivores less abundant in Hainich and Schorfheide, respectively (Supplementary Figure S4A). Soil types significantly influenced the relative abundances of bacterivores and omnivores; bacterivores were most abundant in Leptosol and less abundant in Stagnosol; omnivores were most abundant in Leptosol and Cambisol and less abundant in Histosol and Gleysol (Supplementary Figure S4B). The relative abundance of plant parasites increased with more intense grassland management (from pastures to mowed meadows and meadows) (Supplementary Figure S4C) and higher land use intensity (Supplementary Figure S4D), but decreased with higher C/N ratio (Supplementary Figure S4E). Omnivores and eukaryvores were more abundant at low land use intensity (Supplementary Figure S4D) and high C/N ratio (Supplementary Figure S4E).

In forest, the patterns shown by bacterivores and eukaryvores were opposite: factors favoring one functional group impaired the other – with omnivores showing a somewhat intermediate trend (Supplementary Figure S5). While bacterivores were markedly more abundant in Schorfheide, eukaryvores showed the opposite trend, and the relative abundances of omnivores differed only slightly between the three regions (Supplementary Figure S5A). All trophic guilds were differentially affected by soil type (Supplementary Figure S5B). The identity of the dominant tree species had a strong effect on bacterivores, which were less abundant under beech and spruce than pine and oak – while the opposite was true for eukaryvores (Supplementary Figure S5C). Edaphic factors had a much stronger influence on trophic guilds in forests than in grasslands. The relative abundance of bacterivores was highest at low soil organic carbon content, while the eukaryvores showed the opposite trend; omnivores only slightly increased at a high organic carbon content (Supplementary Figure S5D). Bacterivores increased and eukaryvores decreased at low pH (3–4), while omnivores slightly increased at high pH (Supplementary Figure S5E). Increasing soil organic carbon, pH, clay content and decreased soil C/N ratio all depleted the bacterivores and increased the eukaryvores (Supplementary Figures S5D–G).

## Discussion

Our study was based on a thorough sampling of protists across three regions spanning a South-North gradient in Germany. Sequencing depth reached saturation (Supplementary Figure S2), a precondition to detect detailed responses of each functional group to the ecological processes involved in shaping their distribution. The high percentage of OTUs not closely matching any published sequence (Supplementary Figure S1) indicated a significant hidden species richness not yet taxonomically recorded or sequenced. Consistently with previous studies on Cercozoa and Endomyxa, a high alpha-diversity and a low beta-diversity were found (Lentendu et al., 2018; Fiore-Donno et al., 2019). Our data not only confirmed the low endemicity of Cercozoa, but due to our thorough sampling, high sequencing depth and the use of taxon-specific primers, also showed that almost all OTUs were shared between ecosystems and regions. This implies that community assembly of these protists is not limited by dispersal over countrywide distances. As a corollary, the remarkable differences in beta diversity then must reflect differences in how taxonomic groups and trophic guilds respond to environmental selection (Fiore-Donno et al., 2019).

### Phytomyxea Are Absent From Forests

One remarkable exception to the ubiquitous occurrence of our taxa was the endomyxan plant parasites (Phytomyxea) which, apart from three OTUs, were not represented in the forest ecosystem (Figure 2 and Supplementary Table S6). To our knowledge, this striking pattern was not observed to date, although Ferreira de Araujo et al. (2018) noted that grass-dominated ecosystems hosted more plant parasites than tree-dominated ones. The absence of Phytomyxea in temperate forests is corroborated by a recent metatranscriptomics study on leaf litter of 18 Biodiversity Exploratories forest sites (Voss et al., 2019), however, they were detected in low abundance in tropical forest soils (Mahé et al., 2017). The dominant endomyxan OTUs in grasslands were identified as *Polymyxa graminis*, *Spongospora nasturtii*, and *Woronina pythii* (Supplementary Table S5). The first two are plant parasites associated with grasses and Brassicaceae, respectively. Since both grasses and Brassicaceae are present in forests, although to a lesser extent than in grasslands, the remarkable absence of Phytomyxea from the 300 forest soils cannot be attributed to the lack of suitable hosts. Instead, it might be hypothesized that the establishment of the parasites depends on a threshold density of their host plants, or that the forest soil microbial community has a suppressing effect on Phytomyxea. Both hypotheses deserve further attention due to their potential value for sustainable agriculture. *Woronina pythii* is an endomyxan hyperparasite of *Phytium*, a genus of parasitic oomycetes, belonging to the supergroup Stramenopiles. Our study confirmed that *Woronina* is widespread and relatively abundant in soils (Neuhauser et al., 2014; Fiore-Donno et al., 2019), suggesting a potential use as a biological pest control.

### Responses to Human-Induced Disturbances in Grasslands

The most parsimonious model suggested that both relative abundance and beta diversity of endomyxan plant parasites in grasslands were influenced by management intensity (pastures, mowed meadows, meadows) and the region-dependent soil types (Supplementary Table S6), but not by other edaphic factors. Their relative abundances increased with more intense grassland management (from pasture to meadows) (Supplementary Figure S4C), a higher LUI index (Supplementary Figure S4D) and corresponding shifts toward higher soil nutrient contents (lower C/N, 7–10) (Supplementary Figure S4E). In the Biodiversity Exploratories, the LUI index mainly depended on N fertilization and mowing (Blüthgen et al., 2012). Thus nitrogen-rich, intensively managed meadows constitute an important natural reservoir for these plant parasites. Beta diversity of cercozoan and endomyxan OTUs was influenced by anthropogenic changes such as land use intensification (LUI index), fertilization, and differences in the soil C/N ratio. This confirms a recent study where protists were identified as the most susceptible component of the soil microbiome to the application of nitrogen fertilizers in agricultural soils (Zhao et al., 2019). Mowing intensity had a pronounced negative effect on plant species richness in Hainich (Socher et al., 2013). Accordingly, the significant influence of mowing intensity on protistan beta diversity in Hainich (Supplementary Table S6) was likely driven by shifts in plant species composition.

### Opposite Responses of Trophic Guilds in Forest

**FIGURE 4:**
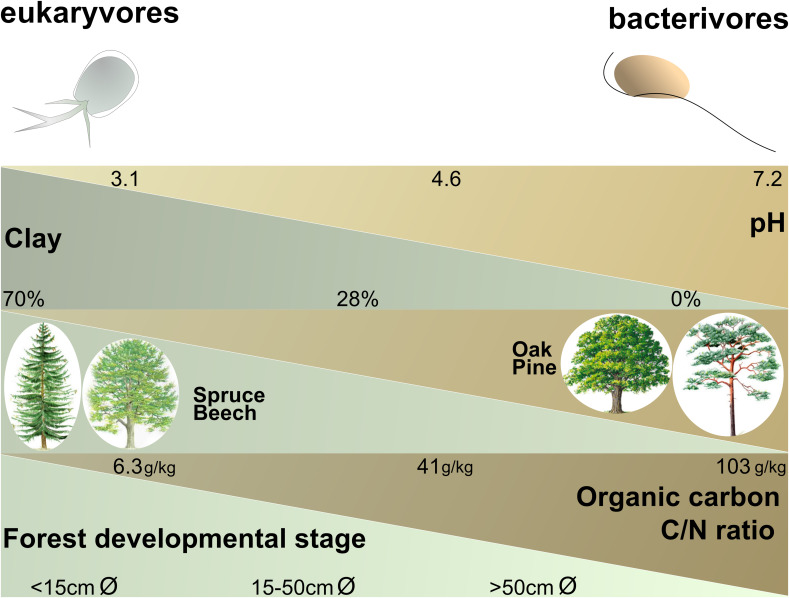
Schematic illustration showing the positive or negative influences of the most influential ecological and edaphic parameters in forest (according to the models in Supplementary Table S6) and the relative abundances of the cercozoan bacterivores and eukaryvores. Minimum, average and maximum values are given for each parameter. Forest developmental stage was estimated by the mean of the diameter of 100 trunks of well-developed trees of the dominant species. The parameters are not co-correlated: e.g., bacterivores are favored by soils with a higher pH or by soils with a higher organic carbon content (not necessarily by both).

Bacterivores and eukaryvores showed opposite responses in their relative abundances to different environmental parameters in forest (Supplementary Figure S5 and Figure 4). Omnivores did not respond so markedly, probably because they combine bacterivory and eukaryvory. Since bacterivores and eukaryvores rely on entirely different food sources, their opposite responses may reflect shifts in the availability of bacteria and microbial eukaryotes linked to specific forest soil conditions. For example, the increase of bacterivores and decrease of eukaryvores in Schorfheide could be explained by the lower biomass of soil fungi in this region (Birkhofer et al., 2012). Bacterivores and eukaryvores were differentially influenced by three edaphic factors, i.e., pH, texture and organic carbon content (Figure 4), the very same major drivers of bacterial community structure at national and continental scales (Bahram et al., 2018; Karimi et al., 2018; Plassart et al., 2019).

### Soil Type and C/N Ratio as Major Structuring Edaphic Forces of Protistan Beta Diversity

Generally, soil type appeared as a major determinant of differences in cercozoan and endomyxan beta diversity between the three regions, confirming previous observations using microbial phospho-lipid fatty acids (PLFAs) (Herold et al., 2014). Soil types also influenced beta diversity within and relative proportions between different trophic guilds, including bacterivores, omnivores and eukaryvores (Figure 4 and Supplementary Figure S5). The importance of soil type is corroborated by significant effects of soil physico-chemical parameters (soil pH, clay content, soil organic matter content) on the regional scale (Supplementary Table S6 and Supplementary Figure S5). Soil types, with their specific physical and chemical properties, are considered to be strong environmental filters for the assembly of microbial communities, sometimes more influential than plant species (Garbeva et al., 2004; Harkes et al., 2019). Importantly, soil types may contribute to explain regional differences in microbial community composition.

Soil C/N ratio was identified as a robust structuring factor of belowground biota across biomes (Fierer et al., 2009), and increasing C/N ratio had been shown to decrease microbial biomass (Dequiedt et al., 2011; Serna-Chavez et al., 2013). In our study sites, C/N ratios were lower in grasslands (7–15) than in forests (11–26) (Supplementary Figures S4, S5). Unexpectedly, while eukaryvores followed the expected pattern of microbial biomass, we found bacterivores to be favored by a high C/N ratio in forest, although such substrates would limit the biomass of bacteria.

Regional differences among the Biodiversity Exploratories have already been observed: the three regions differ substantially in diversity of macro organisms, bacteria, environmental conditions (Allan et al., 2015; Kaiser et al., 2016) and in the main edaphic factors (pH, organic C, clay, C/N ratio) (Supplementary Table S3). Different assemblages of protists reflected differences between the three regions (Glaser et al., 2015; Fiore-Donno et al., 2016), except in the study of Venter et al. (2017), attributed by the authors to under sampling. In our study, the regional differences were more pronounced in forests than in grasslands (Figure 3 and Supplementary Figures S4A, S5A), with Schorfheide standing out. In particular, the clear differences in the abundances of bacterivores and eukaryvores in Schorfheide were mirrored in the types of soil and trees only present in this region (Supplementary Figure S5).

### Strong Differences in Protistan Community Composition Between Grassland and Forest

The main driver shaping the cercozoan and endomyxan communities was the ecosystem, with striking differences between grassland and forest sites in terms of alpha- and beta-diversity, as shown by Shannon index, evenness, principal component analysis and variance partitioning (Supplementary Figure S3 and Figure 3). Similar to our results, a higher richness in forests compared to grasslands and/or agricultural fields and shrublands was reported for protists (albeit not for Cercozoa) (Ferreira de Araujo et al., 2018) and microbial eukaryotes in general (Zhao et al., 2018). A similar trend was observed in the Biodiversity Exploratories for bacteria (Nacke et al., 2001; Foesel et al., 2014; Kaiser et al., 2016) and fungi, both arbuscular mycorrhizal and saprophytic (Birkhofer et al., 2012).

In our settings, the year of sampling only explained a negligible proportion of the variance, probably because in both years sampling took place in April. Thus, the seasonal dependence of protistan communities collected across one year of sampling in one of the investigated grassland plots in Alb (Fiore-Donno et al., 2019), could not be observed.

## Conclusion

Our intensive study on temperate grasslands and forests was based on an established protocol for metabarcoding of two important protistan lineages, classified into ecologically meaningful guilds, which allowed distinguish important trends in community dynamics and biogeographies. Such trends could be masked in studies considering only high-ranking groups of microorganisms, since opposite responses of different trophic guilds – as shown in this study – could add up to a neutral response. Our data identified management practices favoring plant parasites in grassland, mostly linked to N fertilization. Further investigations on co-occurrences of protistan trophic guilds with bacteria and fungi may better elucidate consumer-resource relationships in soil food webs of grassland and forest ecosystems.

## Data Availability Statement

The datasets presented in this study can be found in online repositories. The names of the repository/repositories and accession number(s) can be found at: https://www.ncbi.nlm.nih.gov/genbank/, SRR8404338-41; https://www.ncbi.nlm.nih.gov/genbank/, the 2,101 OTUs (representative sequences) MN322900–MN325000.

## Author Contributions

MB and AF-D conceived the PATHOGEN project. MB administrated the project. AF-D conducted the amplifications, Illumina sequencing and bioinformatics pipeline. AF-D and TR-H performed statistics. AF-D, TR-H, and MB interpreted the data. AF-D wrote the manuscript. All co-authors participated in the revisions and approved the final version of the manuscript.

## Conflict of Interest

The authors declare that the research was conducted in the absence of any commercial or financial relationships that could be construed as a potential conflict of interest.

## Footnotes

1 http://sankey-diagram-generator.acquireprocure.com/

2 https://inkscape.org/en/

## References

[B1] Allan E. Manning P. Alt F. Binkenstein J. Blaser S. Bluthgen N. (2015). Land use intensification alters ecosystem multifunctionality via loss of biodiversity and changes to functional composition. *Ecol. Lett.* 18 834–843. 10.1111/ele.12469 26096863 PMC4744976

[B2] Bahram M. Hildebrand F. Forslund S. K. Anderson J. L. Soudzilovskaia N. A. Bodegom P. M. (2018). Structure and function of the global topsoil microbiome. *Nature* 560 233–237. 10.1038/s41586-018-0386-6 30069051

[B3] Bass D. Tikhonenkov D. V. Foster R. Dyal P. Janouškovec J. Keeling P. J. (2018). Rhizarian ‘Novel Clade 10’ revealed as abundant and diverse planktonic and terrestrial flagellates, including *Aquavolon* n. gen. *J. Eukary. Microbiol.* 65 828–842. 10.1111/jeu.12524 29658156 PMC6282753

[B4] Bass D. Ward G. Burki F. (2019). Ascetosporea. *Curr. Biol.* 29 R1–R15. 10.1016/j.cub.2018.11.025 30620917

[B5] Birkhofer K. Schöning I. Alt F. Herold N. Klarner B. Maraun M. (2012). General relationships between abiotic soil properties and soil biota across spatial scales and different land-use types. *PLoS One* 7:e43292. 10.1371/journal.pone.0043292 22937029 PMC3425568

[B6] Bjorbækmo M. F. M. Evenstad A. Røsæg L. L. Krabberød A. K. Logares R. (2019). The planktonic protist interactome: where do we stand after a century of research? *ISME J.* 14 544–559. 10.1101/587352 31685936 PMC6976576

[B7] Blüthgen N. Dormann C. F. Prati D. Klaus V. H. Kleinebecker T. Hölzel N. (2012). A quantitative index of land-use intensity in grasslands: integrating mowing, grazing and fertilization. *Basic Appl. Ecol.* 13 207–220. 10.1016/j.baae.2012.04.001

[B8] Blüthgen N. Simons N. K. Jung K. Prati D. Renner S. Boch S. (2016). Land use imperils plant and animal community stability through changes in asynchrony rather than diversity. *Nat. Commun.* 7:10697. 10.1038/ncomms10697 26869180 PMC4754335

[B9] Bonkowski M. Dumack K. Fiore-Donno A. M. (2019). “The protists in soil – a token of untold eukaryotic diversity,” in *Modern Soil Microbiology*, eds van Elsas J. D. Trevors J. T. Soares Rosado A. Nannipieri P. (Boca Raton, FL: CRC Press), 125–140. 10.1201/9780429059186-8

[B10] Burki F. Keeling P. J. (2014). Rhizaria. *Curr. Biol.* 24 R103–R107. 10.1016/j.cub.2013.12.025 24502779

[B11] Camacho C. Coulouris G. Avagyan V. Ma N. Papadopoulos J. Bealer K. (2008). BLAST+: architecture and applications. *BMC Bioinform.* 10:421. 10.1186/1471-2105-10-421 20003500 PMC2803857

[B12] Cavalier-Smith T. (1998). A revised six-kingdom system of life. *Biol. Rev.* 73 203–266. 10.1111/j.1469-185X.1998.tb00030.x 9809012

[B13] Cavalier-Smith T. Chao E. E. Lewis R. (2018). Multigene phylogeny and cell evolution of chromist infrakingdom Rhizaria: contrasting cell organisation of sister phyla Cercozoa and Retaria. *Protoplasma* 255 1517–1574. 10.1007/s00709-018-1241-1 29666938 PMC6133090

[B14] de Vries F. T. Thébault E. Liiri M. Birkhofer K. Tsiafouli M. A. Bjørnlund L. (2014). Soil food web properties explain ecosystem services across European land use systems. *Proc. Natl. Acad. Sci. U.S.A.* 110 14296–14301. 10.1073/pnas.1305198110 23940339 PMC3761618

[B15] del Campo J. Kolisko M. Boscaro V. Santoferrara L. F. Nenarokov S. Massana R. (2018). EukRef: phylogenetic curation of ribosomal RNA to enhance understanding of eukaryotic diversity and distribution. *PLoS Biol.* 16:e2005849. 10.1371/journal.pbio.2005849 30222734 PMC6160240

[B16] Dequiedt S. Saby N. P. Lelievre M. Jolivet C. Thioulouse J. Toutain B. (2011). Biogeographical patterns of soil molecular microbial biomass as influenced by soil characteristics and management. *Glob. Ecol. Biogeogr.* 20 641–652. 10.1111/j.1466-8238.2010.00628.x

[B17] Dumack K. Fiore-Donno A. M. Bass D. Bonkowski M. (2019). Making sense of environmental sequencing data: ecologically important functional traits of the protistan groups Cercozoa and Endomyxa (Rhizaria). *Mol. Ecol. Resour.* 20 398–403. 10.1111/1755-0998.13112 31677344

[B18] Edgar R. C. Haas B. Clemente J. C. Quince C. Knight R. (2011). UCHIME improves sensitivity and speed for chimera detection. *Bioinformatics* 27 2194–2200. 10.1093/bioinformatics/btr381 21700674 PMC3150044

[B19] Egorov E. Prati D. Durka W. Michalski S. Fischer M. Schmitt B. (2014). Does land-use intensification decrease plant phylogenetic diversity in local grasslands? *PLoS One* 9:e103252. 10.1371/journal.pone.0103252 25061934 PMC4111588

[B20] Esling P. Lejzerowicz F. Pawlowski J. (2015). Accurate multiplexing and filtering for high-throughput amplicon-sequencing. *Nucl. Acids Res.* 43 2513–2524. 10.1093/nar/gkv107 25690897 PMC4357712

[B21] Ferreira de Araujo A. S. Mendes L. W. Lemos L. N. Lopes Antunes J. E. Aguiar Beserra J. E. Jr. do Carmo Catanho Pereira de Lyra M. (2018). Protist species richness and soil microbiome complexity increase towards climax vegetation in the Brazilian Cerrado. *Commun. Biol.* 1:135. 10.1038/s42003-018-0129-0 30272014 PMC6127325

[B22] Fierer N. Strickland M. S. Liptzin D. Bradford M. A. Cleveland C. C. (2009). Global patterns in belowground communities. *Ecol. Lett.* 12 1–12. 10.1111/j.1461-0248.2009.01360.x 19674041

[B23] Fiore-Donno A. M. Richter-Heitmann T. Degrune F. Dumack K. Regan K. M. Marhan S. (2019). Functional traits and spatio-temporal structure of a major group of soil protists (Rhizaria: Cercozoa) in a temperate grassland. *Front. Microbiol.* 10:1332. 10.3389/fmicb.2019.01332 31244819 PMC6579879

[B24] Fiore-Donno A. M. Rixen C. Rippin M. Glaser K. Samolov E. Karsten U. (2018). New barcoded primers for efficient retrieval of cercozoan sequences in high-throughput environmental diversity surveys, with emphasis on worldwide biological soil crusts. *Mol. Ecol. Resour.* 18 229–239. 10.1111/1755-0998.12729 29058814

[B25] Fiore-Donno A. M. Weinert J. Wubet T. Bonkowski M. (2016). Metacommunity analysis of amoeboid protists in grassland soils. *Sci. Rep.* 6:19068. 10.1038/srep19068 26750872 PMC4707496

[B26] Fischer M. Bossdorf O. Gockel S. Hänsel F. Hemp A. Hessenmöller D. (2010). Implementing largescale and longterm functional biodiversity research: the biodiversity exploratories. *Basic Appl. Ecol.* 11 473–485. 10.1016/j.baae.2010.07.009

[B27] Foesel B. U. Nägele V. Naether A. Wüst P. K. Weinert J. Bonkowski M. (2014). Determinants of *Acidobacteria* activity inferred from the relative abundances of 16S rRNA transcripts in German grassland and forest soils. *Environ. Microbiol.* 16 658–675. 10.1111/1462-2920.12162 23802854

[B28] Frank D. N. (2009). BARCRAWL and BARTAB: software tools for the design and implementation of barcoded primers for highly multiplexed DNA sequencing. *BMC Bioinform.* 10:362. 10.1186/1471-2105-10-362 19874596 PMC2777893

[B29] Garbeva P. van Veen J. Van Elsas J. D. (2004). Microbial diversity in soil: selection of microbial populations by plant and soil type and implications for disease suppressiveness. *Annu. Rev. Phytopathol.* 42 243–270. 10.1146/annurev.phyto.42.012604.135455 15283667

[B30] Geisen S. Koller R. Hünninghaus M. Dumack K. Urich T. Bonkowski M. (2016). The soil food web revisited: diverse and widespread mycophagous soil protists. *Soil Biol. Biochem.* 94 10–18. 10.1016/j.soilbio.2015.11.010

[B31] Geisen S. Tveit A. Clark I. M. Richter A. Svenning M. M. Bonkowski M. (2015). Metatranscriptomic census of active protists in soils. *ISME J.* 9 2178–2190. 10.1038/ismej.2015.30 25822483 PMC4579471

[B32] Glaser K. Kuppardt A. Boenigk J. Harms H. Fetzer I. Chatzinotas A. (2015). The influence of environmental factors on protistan microorganisms in grassland soils along a land-use gradient. *Sci. Total Environ.* 537 33–42. 10.1016/j.scitotenv.2015.07.158 26282737

[B33] Grossmann L. Jensen M. Heider D. Jost S. Glücksman E. Hartikainen H. (2016). Protistan community analysis: key findings of a large-scale molecular sampling. *ISME J.* 10 2269–2279. 10.1038/ismej.2016.10 26859769 PMC4989302

[B34] Guillou L. Bachar D. Audic S. Bass D. Berney C. Bittner L. (2013). The Protist Ribosomal Reference database (PR2): a catalog of unicellular eukaryote small subunit rRNA sequences with curated taxonomy. *Nucl. Acids Res.* 41 D597–D604. 10.1093/nar/gks1160 23193267 PMC3531120

[B35] Harkes P. Suleiman A. K. A. van den Elsen S. J. J. de Haan J. J. Holterman M. Kuramae E. E. (2019). Conventional and organic soil management as divergent drivers of resident and active fractions of major soil food web constituents. *Sci. Rep.* 9:13521. 10.1038/s41598-019-49854-y 31534146 PMC6751164

[B36] Hassani A. M. Durán P. Hacquard S. (2018). Microbial interactions within the plant holobiont. *Microbiome* 6:58. 10.1186/s40168-018-0445-0 29587885 PMC5870681

[B37] Henriksen M. V. Chapple D. G. Chown S. L. McGeoch M. A. (2019). The effect of network size and sampling completeness in depauperate networks. *J. Anim. Ecol.* 88 211–222. 10.1111/1365-2656.12912 30291749

[B38] Herold N. Schöning I. Gutknecht J. Alt F. Boch S. Müller J. (2014). Soil property and management effects on grassland microbial communities across a latitudinal gradient in Germany. *Appl. Soil Ecol.* 73 41–50. 10.1016/j.apsoil.2013.07.009

[B39] Hunt H. Coleman D. C. Ingham E. Elliott E. Moore J. C. Rose S. (1987). The detrital food web in a shortgrass prairie. *Biol. Fertil. Soils* 3 57–68. 10.1007/BF00260580

[B40] Kaiser K. Wemheuer B. Korolkow V. Wemheuer F. Nacke H. Schöning I. (2016). Driving forces of soil bacterial community structure, diversity, and function in temperate grasslands and forests. *Sci. Rep.* 6:33696. 10.1038/srep33696 27650273 PMC5030646

[B41] Karimi B. Terrat S. Dequiedt S. Saby N. P. Horrigue W. Lelièvre M. (2018). Biogeography of soil bacteria and archaea across France. *Sci. Adv.* 4:eaat1808. 10.1126/sciadv.aat1808 29978046 PMC6031370

[B42] Keil D. Meyer A. Berner D. Poll C. Schützenmeister A. Piepho H.-P. (2011). Influence of land-use intensity on the spatial distribution of N-cycling microorganisms in grassland soils. *FEMS Microbiol. Ecol.* 77 95–106. 10.1111/j.1574-6941.2011.01091.x 21410493

[B43] Lanzén A. Epelde L. Blanco F. Martín I. Artetxe U. Garbisu C. (2016). Multi-targeted metagenetic analysis of the influence of climate and environmental parameters on soil microbial communities along an elevational gradient. *Sci. Rep.* 6:28257. 10.1038/srep28257 27321429 PMC4913321

[B44] Lentendu G. Mahé F. Bass D. Rueckert S. Stoeck T. Dunthorn M. (2018). Consistent patterns of high alpha and low beta diversity in tropical parasitic and free-living protists. *Mol. Ecol.* 27 2846–2857. 10.1111/mec.14731 29851187

[B45] Lentendu G. Wubet T. Chatzinotas A. Wilhelm C. Buscot F. Schlegel M. (2014). Effects of long-term differential fertilization on eukaryotic microbial communities in an arable soil: a multiple barcoding approach. *Mol. Ecol.* 23 3341–3355. 10.1111/mec.12819 24888892

[B46] Mahé F. De Vargas C. Bass D. Czech L. Stamatakis A. Lara E. (2017). Parasites dominate hyperdiverse soil protist communities in Neotropical rainforests. *Nat. Ecol. Evol.* 1:0091. 10.1038/s41559-017-0091 28812652

[B47] Michu E. Mráèková M. Vyskot B. Žlùvová J. (2010). Reduction of heteroduplex formation in PCR amplification. *Biol. Plant.* 54 173–176. 10.1007/s10535-010-0029-8

[B48] Mitchell C. E. (2003). Trophic control of grassland production and biomass by pathogens. *Ecol. Lett.* 6 147–155. 10.1046/j.1461-0248.2003.00408.x

[B49] Nacke H. Thürmer A. Wollherr A. Will C. Hodac L. Herold N. (2001). Pyrosequencing-based assessment of bacterial community structure along different management types in German forest and grassland soils. *PLoS One* 6:e17000. 10.1371/journal.pone.0017000 21359220 PMC3040199

[B50] Neuhauser S. Kirchmair M. Bulman S. R. Bass D. (2014). Cross-kingdom host shifts of phytomyxid parasites. *BMC Evol. Biol.* 14:33. 10.1186/1471-2148-14-33 24559266 PMC4016497

[B51] Oksanen J. Blanchet F. G. Kindt R. Legendre P. Minchin P. R. O’Hara R. B. (2013). *Vegan**: Community Ecology Package. R package version 2.0-10.* Available at: http://CRAN.R-project.org/package=vegan (accessed July, 2020).

[B52] Oliverio A. M. Geisen S. Delgado-Baquerizo M. Maestre F. T. Turner B. L. Fierer N. (2020). The global-scale distribution of soil protists and their contributions to belowground systems. *Sci. Adv.* 6:eaax8787. 10.1126/sciadv.aax8787 32042898 PMC6981079

[B53] Pawlowski J. Adl S. M. Audic S. Bass D. Belbahri L. Berney C. (2012). CBOL Protist Working Group: barcoding eukaryotic richness beyond the animal, plant and fungal kingdoms. *PLoS Biol.* 10:e1001419. 10.1371/journal.pbio.1001419 23139639 PMC3491025

[B54] Plassart P. Chemidlin Prévost-Bouré N. Uroz S. Dequiedt S. Stone D. Creamer R. (2019). Soil parameters, land use, and geographical distance drive soil bacterial communities along a European transect. *Sci. Rep.* 9:605. 10.1038/s41598-018-36867-2 30679566 PMC6345909

[B55] Potatov A. Brose U. Scheu S. Tiunov A. V. (2019). Trophic position of consumers and size structure of food webs across aquatic and terrestrial ecosystems. *Am. Nat.* 194 823–839. 10.1086/705811 31738104

[B56] R Development Core Team, (2014). *R: A Language and Environment for Statistical Computing.* Vienna: R Foundation for Statistical Computing.

[B57] Reyns W. Rineau F. Spaak J. W. Franken O. Berg M. P. Van Der Plas F. (2019). Food web uncertainties influence predictions of climate change effects on soil carbon sequestration in heathlands. *Microb. Ecol.* 79 686–693. 10.1007/s00248-019-01444-1 31654107

[B58] Rognes T. Flouri T. Nichols B. Quince C. Mahé F. (2016). VSEARCH: a versatile open source tool for metagenomics. *PeerJ* 4:e2584. 10.7717/peerj.2584 27781170 PMC5075697

[B59] Scheu S. (2002). The soil food web: structure and perspectives. *Eur. J. Protistol.* 38 11–20. 10.1016/S1164-5563(01)01117-7

[B60] Schloss P. D. Westcott S. L. Ryabin T. Hall J. R. Hartmann M. Hollister E. B. (2009). Introducing mothur: open-source, platform-independent, community-supported software for describing and comparing microbial communities. *Appl. Environ. Microbiol.* 75 7537–7541. 10.1128/AEM.01541-09 19801464 PMC2786419

[B61] Schnitzer S. A. Klironomos J. HilleRisLambers J. Kinkel L. Reich P. Xiao K. (2011). Soil microbes drive the classic plant diversity–productivity pattern. *Ecology* 92 296–303. 10.1890/10-0773.1/full 21618909

[B62] Schulz G. Schneider D. Brinkmann N. Edy N. Daniel R. Polle A. (2019). Changes in trophic groups of protists with conversion of rainforest into rubber and oil palm plantations. *Front. Microbiol.* 10:240. 10.3389/fmicb.2019.00240 30809219 PMC6380168

[B63] Serna-Chavez H. M. Fierer N. van Bodegom P. (2013). Global drivers and patterns of microbial abundance in soil. *Glob. Ecol. Biogeogr.* 22 1162–1172. 10.1111/geb.12070

[B64] Shen C. Liang W. Shi Y. Lin X. Zhang H. Wu X. (2014). Contrasting elevational diversity patterns between eukaryotic soil microbes and plants. *Ecology* 95 3190–3202. 10.1890/14-0310.1.sm

[B65] Socher S. A. Prati D. Boch S. Müller J. Baumbach H. Gockel S. (2013). Interacting effects of fertilization, mowing and grazing on plant species diversity of 1500 grasslands in Germany differ between regions. *Basic Appl. Ecol.* 14 126–136. 10.1016/j.baae.2012.12.003

[B66] Solly E. F. Schöning I. Boch S. Kandeler E. Marhan S. Michalzik B. (2014). Factors controlling decomposition rates of fine root litter in temperate forests and grasslands. *Plant Soil* 382 203–218. 10.1007/s11104-014-2151-4

[B67] Stukenbrock E. McDonald B. (2008). The origins of plant pathogens in agro-ecosystems. *Annu. Rev. Phytopathol.* 46 75–100. 10.1146/annurev.phyto.010708.154114 18680424

[B68] van der Heijden M. G. Bardgett R. D. van Straalen N. M. (2008). The unseen majority: soil microbes as drivers of plant diversity and productivity in terrestrial ecosystems. *Ecol. Lett.* 11 296–310. 10.1111/j.1461-0248.2007.01139.x 18047587

[B69] Venter P. C. Nitsche F. Domonell A. Heger P. Arndt H. (2017). The protistan microbiome of grassland soil: diversity in the mesoscale. *Protist* 168 546–564. 10.1016/j.protis.2017.03.005 28961455

[B70] Voss C. Fiore-Donno A. M. Guerreiro M. A. Peršoh D. Bonkowski M. (2019). Metatranscriptomics reveal unsuspected protistan diversity in leaf litter across temperate beech forests, with Amoebozoa the dominating lineage. *FEMS Microbiol. Ecol.* 95:fiz142. 10.1093/femsec/fiz142 31557276

[B71] Xiong W. Jousset A. Guo S. Karlsson I. Zhao Q. Wu H. (2018). Soil protist communities form a dynamic hub in the soil microbiome. *ISME J.* 12 634–638. 10.1038/ismej.2017.171 29028001 PMC5776453

[B72] Zhao H. Li X. Zhang Z. Zhao Y. Chen P. Zhu Y. (2018). Drivers and assemblies of soil eukaryotic microbes among different soil habitat types in a semi-arid mountain in China. *PeerJ* 6:e6042. 10.7717/peerj.6042 30568857 PMC6286657

[B73] Zhao Z.-B. He J.-Z. Geisen S. Han L.-L. Wang J.-T. Shen J.-P. (2019). Protist communities are more sensitive to nitrogen fertilization than other microorganisms in diverse agricultural soils. *Microbiome* 7:33. 10.1186/s40168-019-0647-0 30813951 PMC6393985

