# Supplementary material for: Contrasting Responses of Protistan Plant Parasites and Phagotrophs to Ecosystems, Land Management and Soil Properties
Source: Front Microbiol. 2020 Aug 5;11:1823. doi: 10.3389/fmicb.2020.01823 (PMC7422690; doi:10.3389/fmicb.2020.01823)
Supplement: Supplementary file 1 [file Data_Sheet_1.doc]

# Fiore-Donno et al. 2020. Protistan plant pathogens and phagotrophs

# Supplementary information, legends

**Supplementary tables**

**Table S1**. Environmental parameters from the 150 grassland study sites and two years of collection.

**Table S2**. Environmental parameters from the 150 forest study sites and two years of collection.

**Table S3.** Average and standard deviation of the continuous edaphic and environmental variables from Tables S1 & S2, by ecosystem (150 sites each), region (50 sites each) and year of sampling. Significant differences between regions are indicated by a different letter. Regional differences are important, while there is little variation between years.

**Table S4**. Primers and combinations of barcodes used in this study, with the corresponding soil samples. Code for sample names: AE=Alb, HE=Hainich, SE=Schorfheide. G=grassland sites - to be replaced by F in forest sites (the same barcodes were applied since the grassland and forest samples were amplified and sequenced separately).

**Table S5**. Database of the abundance of each cercozoan and endomyxan OTU per sample. The taxonomic assignment (super group, class, order, family, genus and species) is provided according to the best hit by BLAST (PR2 database), with the % of similarity. Functional traits (morphology, nutrition and locomotion modes) were estimated to the genus level following Dumack et al. (2019).

**Table S6**. Most parsimonious models (dbRDA), with their respective R^2^ adjusted and F values, and the F values of the factors selected by each model. Significance values shown as symbol (see footnote).

## Supplementary figures

**Figure S1**. Similarities of the cercozoan and endomyxan OTUs with known sequences. OTUs are classified according to their percentage of similarity to the next kin by BLAST. The horizontal bar length is proportional to the number of OTUs in each rank. Shaded area=OTUs with a similarity ≥ 97%.

**Figure S2**. Description of the cercozoan and endomyxan diversity. A. Rarefaction curve describing the observed number of OTUs as a function of the sequencing effort; saturation was reached with c. 235,000 sequences. B. Species accumulation curve describing the sampling effort; saturation was reached with 65 samples.

**Figure S3**. Boxplots and table of the alpha diversity of the cercozoan and endomyxan OTUs estimated with the Shannon and evenness indices, for all sites and for sites binned by region and ecotype. Red letters: a change from “a” to “b”, or “c” indicates a significant difference (multiple comparison of means, Tukey's test); two or three letters (e.g. “ab” or "abc") indicate non-significant differences between plots sharing those letters. Red dots indicate the means (black lines the median). In the table, the highest means are in bold.

**Figure S4**. Boxplots of the variation of the relative abundances of the four main nutrition modes of Cercozoa and Endomyxa in grassland, coloured according to region. **A.** by region; **B.** by soil type, only Cambisoil is found in the three regions; **C.** by grassland management; **D.** by land use intensity (LUI) index, transformed into a categorical variable according to quantiles. **E**. by C/N ratio, transformed as in D. The y-scale varies between graphs. Red letters: a change from “a” to “b”, or “c” indicates a significant difference (multiple comparison of means, Tukey's test); two or three letters (e.g. “ab” or "abc") indicate non-significant differences between plots sharing those letters. Red lines indicate the mean.

**Figure S5**. Boxplots of the variation of the relative abundances of the three main nutrition modes of Cercozoa in forest (nearly no plant parasites were found in forest), coloured according to region. **A.** by region; **B.** by soil type, only Cambisoil is found in two regions; **C.** by main tree species, Latin names: beech = *Fagus sylvatica*, spruce = *Picea abies*, pine = *Pinus sylvestris*, oak = *Quercus petrea* & *Q. robur*, pine and oak growing only in Schorfheide; **D**. by organic carbon (g/kg soil) levels; **E**. by pH levels; **F**. by levels of percentage of soil clay content. **G**. by levels of C/N ratio. The y-scale varies between graphs. Red letters: a change from “a” to “b”, or “c” indicates a significant difference (multiple comparison of means, Tukey's test); two or three letters (e.g. “ab” or "abc") indicate non-significant differences between boxes sharing those letters. Red lines indicate the mean.
