# Supplementary material for: Contrasting Responses of Protistan Plant Parasites and Phagotrophs to Ecosystems, Land Management and Soil Properties
Source: Front Microbiol. 2020 Aug 5;11:1823. doi: 10.3389/fmicb.2020.01823 (PMC7422690; doi:10.3389/fmicb.2020.01823)

Fig. S1. Similarities of the OTUs with known sequences. OTUs are classified according to their percentage of similarity to the next kin by BLAST. The horizontal bar lentgh is proportional to the number of OTUs in each rank. Shaded area=OTUs with a similarity ≥ 97%.

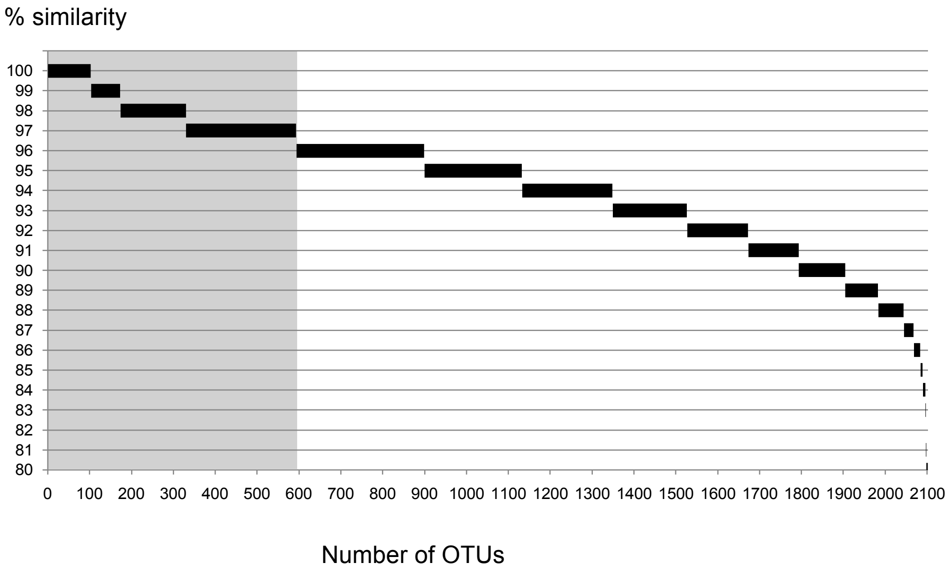

Supplement: Supplementary file 2 [file Data_Sheet_2.zip › Figure S1.PDF]
