# Supplementary material for: Contrasting Responses of Protistan Plant Parasites and Phagotrophs to Ecosystems, Land Management and Soil Properties
Source: Front Microbiol. 2020 Aug 5;11:1823. doi: 10.3389/fmicb.2020.01823 (PMC7422690; doi:10.3389/fmicb.2020.01823)

Figure S2. Description of the diversity. **A**. Rarefaction curve describing the observed number of OTUs as a function of the sequencing effort; saturation was reached with c. 235,000 sequences. **B**. Species accumulation curve describing the sampling effort; saturation was reached with 65 samples.

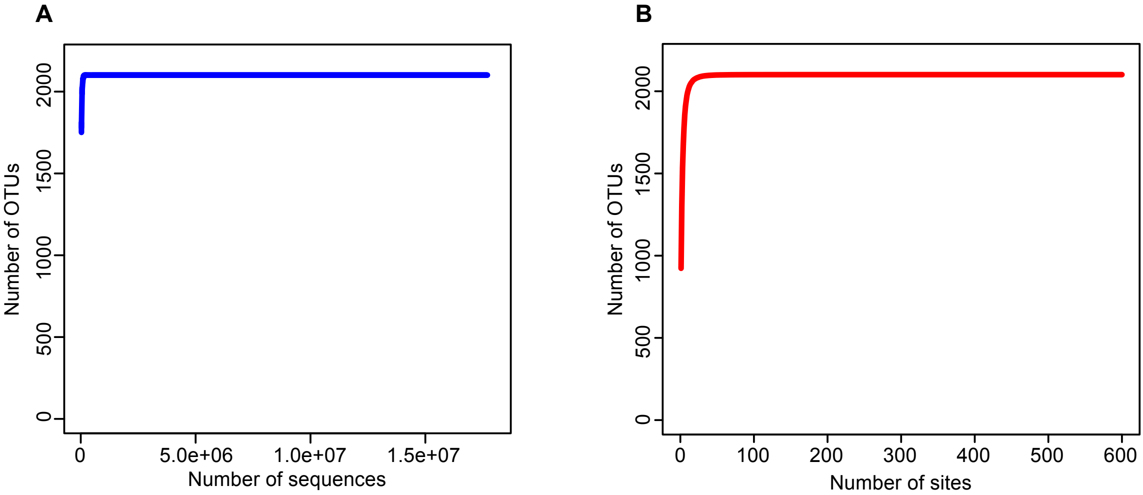

Supplement: Supplementary file 2 [file Data_Sheet_2.zip › Figure S2.PDF]
