# Supplementary material for: Contrasting Responses of Protistan Plant Parasites and Phagotrophs to Ecosystems, Land Management and Soil Properties
Source: Front Microbiol. 2020 Aug 5;11:1823. doi: 10.3389/fmicb.2020.01823 (PMC7422690; doi:10.3389/fmicb.2020.01823)
Supplement: Supplementary file 2 [file Data_Sheet_2.zip › Figure S3.PDF]

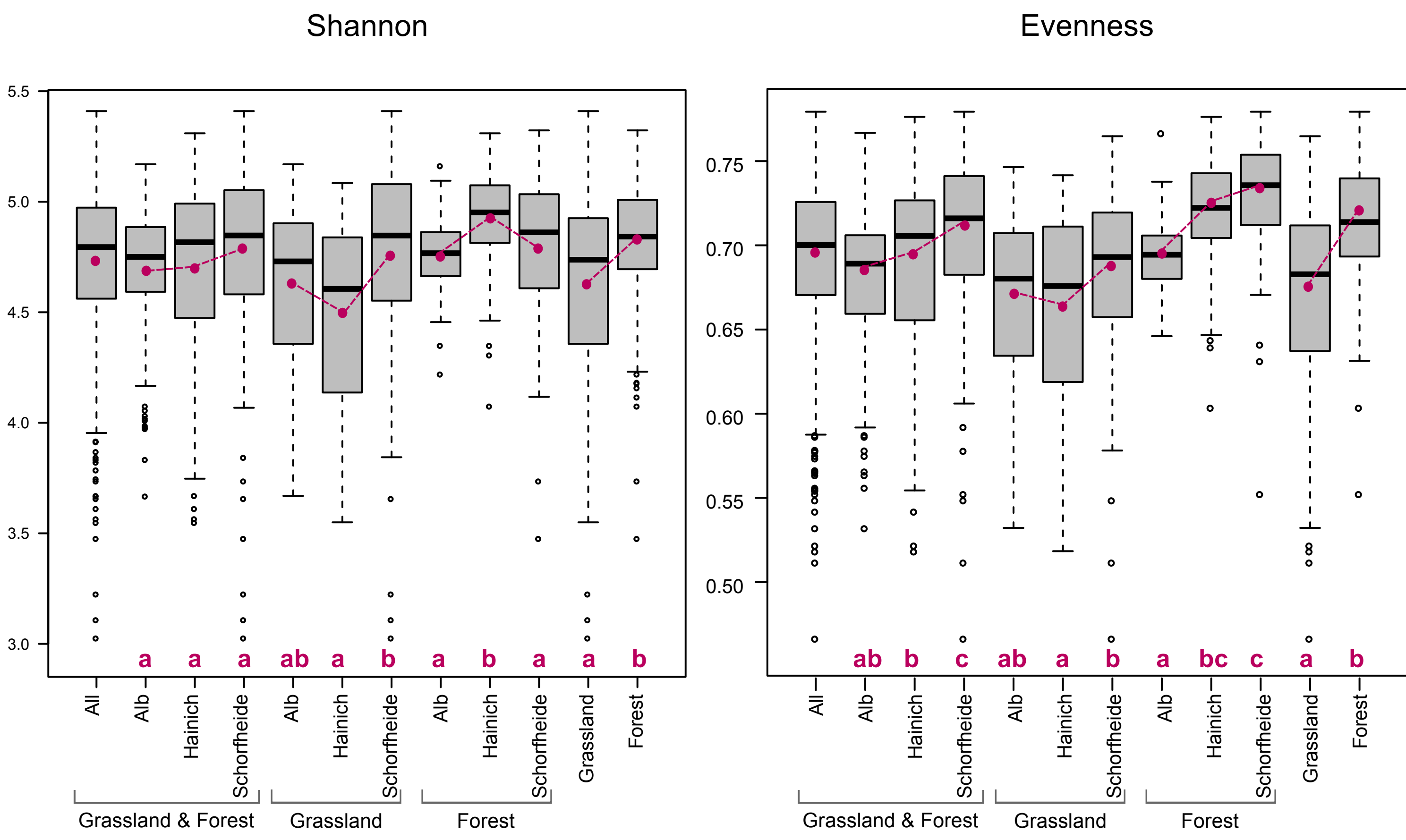

	All sites	Alb	Hainich	Schorfheide	Alb grassland	Hainich grassland	Schorfheide grassland	Alb forest	Hainich forest	Schorfheide forest	Grassland	Forest
**Shannon**												
**Min.**	3.03	3.67	3.55	3.03	3.67	3.55	3.03	4.22	4.08	3.48	3.03	3.48
**Mean**	4.72	4.70	4.70	**4.77**	4.63	4.49	**4.76**	4.76	**4.92**	4.79	4.63	**4.82**
**Max.**	5.41	5.17	5.31	5.41	5.17	5.08	5.41	5.16	5.31	5.32	5.41	5.32
**St_Dev**	0.36	0.27	0.40	0.40	0.34	0.41	0.46	0.17	0.22	0.33	0.42	0.26
**evenness**												
**Min.**	0.47	0.54	0.52	0.47	0.54	0.52	0.47	0.65	0.61	0.56	0.47	0.56
**Mean**	0.70	0.68	0.69	**0.71**	0.67	0.66	**0.69**	0.70	0.72	**0.73**	0.67	**0.72**
**Max.**	0.78	0.77	0.78	0.78	0.75	0.75	0.77	0.77	0.78	0.78	0.77	0.78
**St_Dev**	0.05	0.04	0.06	0.05	0.05	0.06	0.05	0.02	0.03	0.04	0.05	0.03

Figure S3. Boxplots and table of the alpha diversity of the cercozoan and endomyxan OTUs estimated with the Shannon and evenness indices, for all sites and for sites binned by region and ecotype. Red letters: a change from “a” to “b”, or “c” indicates a significant difference (multiple comparison of means, Tukey's test); two or three letters (e.g. “ab” or "abc") indicate non-significant differences between plots sharing those letters. Red dots indicate the means (black lines the median). In the table, the highest means are in bold.
