# Supplementary material for: Contrasting Responses of Protistan Plant Parasites and Phagotrophs to Ecosystems, Land Management and Soil Properties
Source: Front Microbiol. 2020 Aug 5;11:1823. doi: 10.3389/fmicb.2020.01823 (PMC7422690; doi:10.3389/fmicb.2020.01823)
Supplement: Supplementary file 2 [file Data_Sheet_2.zip › Figure S5.PDF]

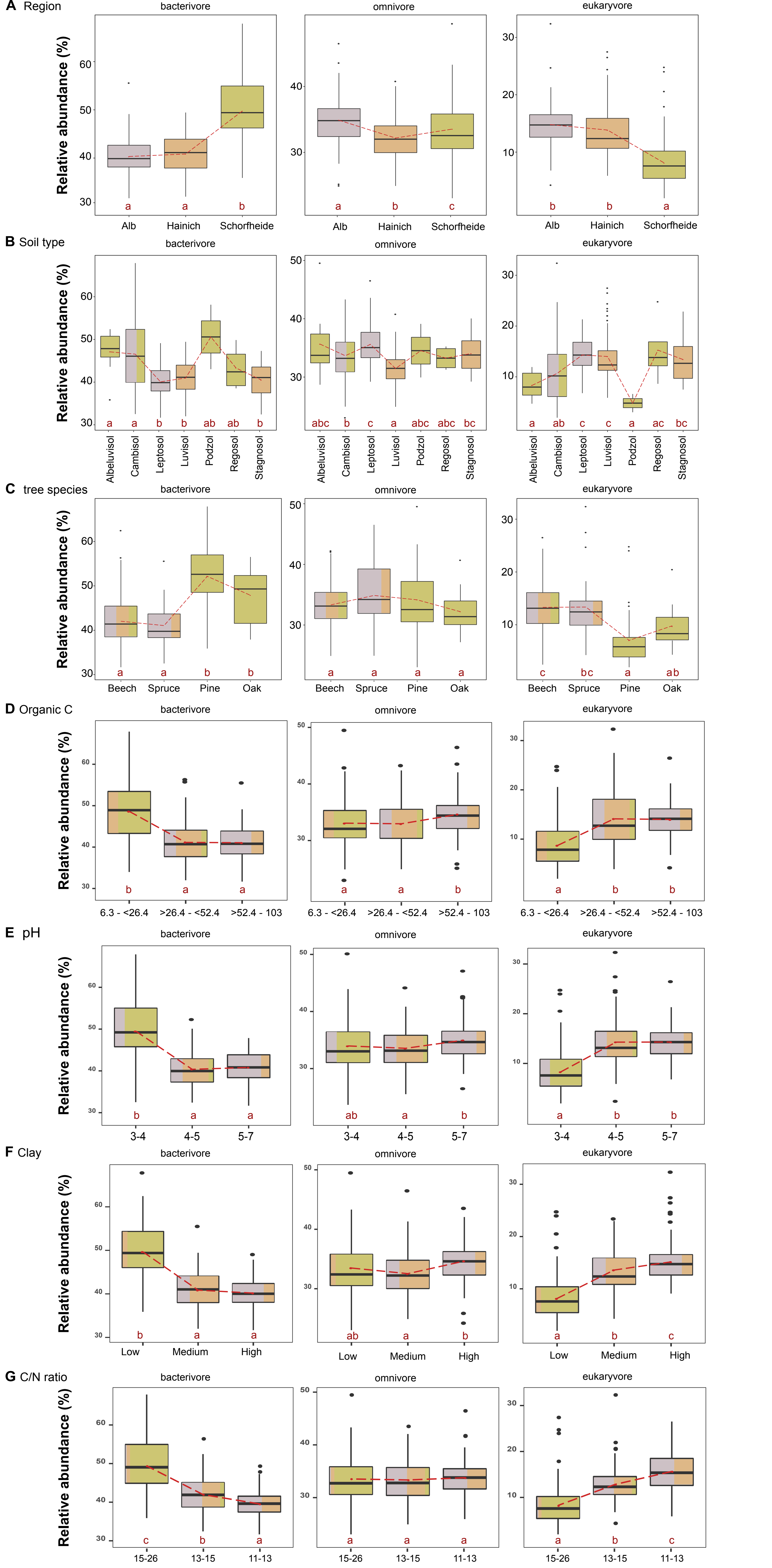

Figure S5. Boxplots of the relative abundances of the three main nutrition modes of Cercozoa in forest (nearly no plant parasites were found in forest), coloured according to region. **A**. by region; **B**. by soil type, only Cambisoil is found in two regions; **C**. by main tree species, Latin names: beech = *Fagus sylvatica*, spruce = *Picea abies*, pine = *Pinus sylvestris*, oak = *Quercus petrea* & *Q. robur*, pine and oak growing only in Schorfheide; **D**. by organic carbon (g/kg soil) levels; **E**. by pH levels; **F.** by levels of percentage of soil clay content; **G**. by levels of C/N ratio. The y-scale varies between graphs. Red letters: a change from "a" to "b", or "c" indicates a significant difference (multiple comparison of means, Tukey's test); two or three letters (e.g. "ab" or "abc") indicate non-significant differences between boxes sharing those letters. Red lines indicate the mean. Right side, tables with regional repartition of each factor; 100 sites in Alb, 95 in Hainich and 97 in Schorfheide.
