# Supplementary material for: Contrasting Responses of Protistan Plant Parasites and Phagotrophs to Ecosystems, Land Management and Soil Properties
Source: Front Microbiol. 2020 Aug 5;11:1823. doi: 10.3389/fmicb.2020.01823 (PMC7422690; doi:10.3389/fmicb.2020.01823)
Supplement: Supplementary file 3 [file Data_Sheet_3.zip › Table S1.pdf]

Table S1. Environmental parameters from the 150 grassland study sites and two years of collection.

Reference dataset at https://www.bexis.uni-jena.de/	14447 22246	14446 23846	14446 23846	14446 23846	14446 23846	14446 23846	14686	14686	14686	19266				10580
Sites (AE=Alb, HE=Hainich, SE=Schorfheide, 11/17:year of collection	pH	Total_C g/kg soil	Inorganic_C g/kg	Organic_C g/kg	Total_N g/kg	CN_ratio	Clay	Silt	Sand	LUI	Grazing[1]	Mowing[2]	Fertilization (kg N/ha)	soil_type
AEG001_11	6.53	85.95	0.96	84.98	8.71	9.76	643	324	33	1.87	0	2	92.75	Leptosol
AEG002_11	6.75	72.92	1.87	71.04	7.49	9.48	512	411	77	3.21	0	3	239	Leptosol
AEG003_11	6.22	59.81	0.28	59.53	5.83	10.21	672	298	30	1.94	0	2	64.6	Leptosol
AEG004_11	5.08	44.53	0.22	44.31	4.83	9.17	488	427	85	1.56	47.4	1	35	Leptosol
AEG005_11	6.14	90.77	0.57	90.19	8.72	10.34	365	518	117	1.52	37	1	35	Leptosol
AEG006_11	5.87	83.14	0.4	82.74	8.21	10.07	588	380	32	1.78	128	1	35	Leptosol
AEG007_11	7.11	86.32	33.51	52.8	4.64	11.37	385	427	188	0.63	42.9	0	0	Leptosol
AEG008_11	6.52	86.63	0.48	86.14	7.68	11.21	673	319	8	1.4	119	1	0	Leptosol
AEG009_11	6.7	71.21	0.93	70.27	5.9	11.91	556	417	27	0.9	85.5	0	0	Leptosol
AEG010_11	6.08	94.16	0.45	93.71	9.1	10.29	647	327	26	0.91	0	1	0	Leptosol
AEG011_11	5.49	62.91	0.31	62.61	6.02	10.39	643	327	30	2.05	0	3	101.76	Leptosol
AEG012_11	6.57	69.15	1.62	67.54	6.59	10.25	436	512	52	1.87	0	2	200.55	Leptosol
AEG013_11	5.95	68.88	0.44	68.43	6.58	10.39	643	311	46	1.87	0	2	200.55	Leptosol
AEG014_11	6.71	62.33	1.06	61.27	6.07	10.09	521	453	26	2.14	0	2	89.6	Leptosol
AEG015_11	5.68	63.9	0.28	63.62	6.51	9.77	642	340	18	2.63	0	3	135.66	Leptosol
AEG016_11	6.61	60.7	0.72	59.98	5.93	10.11	528	449	23	1.57	83.7	2	0	Leptosol
AEG017_11	6.86	67.23	2.55	64.68	6.54	9.89	618	365	17	2.08	0	2	80.75	Leptosol
AEG018_11	6.87	66.09	3.94	62.14	6.31	9.84	649	332	19	2.11	0	2	84.6	Leptosol
AEG019_11	5.85	78.05	0.38	77.67	7.64	10.17	637	327	36	1.61	65.1	1	35	Leptosol
AEG020_11	6.62	81.25	0.84	80.41	8.64	9.31	597	334	69	1.81	350	0	0	Leptosol
AEG021_11	5.99	67.75	0.34	67.42	6.76	9.98	587	391	22	3.72	1226	1	7.95	Leptosol
AEG022_11	5.88	53.56	0	53.56	4.8	11.16	708	270	22	1.08	35.1	1	106.48	Leptosol
AEG023_11	6.77	83.43	3.16	80.28	8.17	9.83	566	387	47	0.91	0	1	0	Leptosol
AEG024_11	6.08	80.7	0.47	80.23	7.78	10.31	638	329	33	1.84	164	2	47.3	Leptosol
AEG025_11	6.97	99.27	17.13	82.13	7.29	11.27	463	406	131	0.76	61.9	0	47.3	Leptosol
AEG026_11	6.66	70.02	0.6	69.42	5.73	12.12	679	241	80	1.81	350	0	0	Leptosol
AEG027_11	6.15	89.96	0.49	89.47	7.69	11.63	576	410	14	1.2	153	0	0	Leptosol
AEG028_11	6.08	54.34	0.31	54.04	4.83	11.19	599	385	16	0.83	73.1	0	0	Leptosol
AEG029_11	5.77	53.67	0.3	53.37	5.17	10.32	566	343	91	1.36	66	1	19.8	Cambisol
AEG030_11	6.65	65.91	0.95	64.96	6.68	9.73	556	375	69	1.79	254	1	94.6	Leptosol
AEG031_11	6.55	70.21	0.58	69.63	6.83	10.18	595	381	24	1.95	318	1	0	Leptosol
AEG032_11	5.3	42.05	0.26	41.79	3.93	10.64	461	466	73	0.75	59.6	0	0	Leptosol
AEG033_11	5.98	49.21	0.28	48.93	4.61	10.61	597	388	15	1.32	186	0	0	Leptosol
AEG034_11	6.17	46.23	0.33	45.9	4.72	9.72	423	554	23	1.02	111	0	0	Leptosol
AEG035_11	5.67	39.54	0	39.54	4.03	9.81	446	503	51	1.83	0	2	45	Cambisol
AEG036_11	6.02	53.74	0.42	53.33	5.14	10.38	588	213	199	1.84	0	2	101.76	Cambisol
AEG037_11	6.1	63.43	0.43	63	6.31	9.99	566	334	100	1.68	0	2	35	Cambisol
AEG038_11	5.51	49.45	0	49.45	5.13	9.64	451	468	81	1.29	0	2	0	Cambisol
AEG039_11	5.67	66.41	0.32	66.09	6.28	10.52	463	493	44	1.68	0	2	35	Cambisol
AEG040_11	6.71	69.95	1.6	68.34	7.02	9.74	698	269	33	1.64	0	3	47.3	Cambisol
AEG041_11	6.15	44.56	0.28	44.28	4.31	10.26	541	407	52	2.25	26	2	117.84	Cambisol
AEG042_11	6.97	96.45	13.31	83.13	9.23	9	159	757	84	2.14	85	2	64.6	Cambisol
AEG043_11	6.81	75.63	3.06	72.57	7.47	9.71	613	349	38	1.73	150	0	48.45	Cambisol
AEG044_11	7.05	78.49	14.47	64.01	6.65	9.63	152	735	113	2.18	504	0	0	Cambisol

AEG045_11	5.19	55.07	0.2	54.86	5.72	9.6	529	417	54	1.29	0	2	0	Cambisol
AEG046_11	5.68	71.91	0.23	71.68	7.25	9.88	659	287	54	1.66	293	0	0	Cambisol
AEG047_11	7.19	104.4	45.38	59.05	5.47	10.8	146	704	150	0.81	69.3	0	0	Cambisol
AEG048_11	7.3	97.98	54.58	43.39	3.38	12.84	295	565	140	0.75	59.1	0	0	Cambisol
AEG049_11	6.16	50.14	0.27	49.87	4.81	10.36	451	522	27	1.05	117	0	0	Cambisol
AEG050_11	5.93	68.98	0.31	68.68	6.76	10.15	589	382	29	1.57	0	2	189.2	Cambisol
HEG001_11	6.65	54.78	0.76	54.02	5.46	9.89	502	447	50	2.42	0	2	150	Cambisol
HEG002_11	7.2	39.23	4.15	35.08	3.55	9.88	552	396	52	2.63	0	3	150	Vertisol
HEG003_11	7.26	39.17	5.88	33.29	3.4	9.81	544	399	57	2.63	0	3	150	Vertisol
HEG004_11	6.65	65.32	0.6	64.72	6.21	10.42	511	414	75	2.18	180	1	81	Stagnosol
HEG005_11	7.09	48.4	4.8	43.6	4.44	9.82	454	497	49	2.61	149	3	90	Stagnosol
HEG006_11	5.96	20.77	0	20.77	2.01	10.33	257	698	45	2.32	107	1	132	Stagnosol
HEG007_11	6.99	57.9	2.41	55.48	5.7	9.73	536	434	30	0.58	36	0	0	Stagnosol
HEG008_11	7.17	60.63	3.59	57.04	5.78	9.86	494	452	56	0.58	36	0	0	Stagnosol
HEG009_11	6.92	42.26	1.14	41.11	3.58	11.49	387	537	76	0.75	59.5	0	0	Stagnosol
HEG010_11	6.4	44.03	0.3	43.74	4.01	10.9	436	532	30	1.59	99.3	1	170.28	Vertisol
HEG011_11	7.25	65.07	8.44	56.64	5.45	10.4	552	405	43	1.46	57.4	1	170.28	Stagnosol
HEG012_11	6.98	78.38	3.49	74.89	7.86	9.53	497	448	55	3.42	1143	0	40	Stagnosol
HEG013_11	7.17	38.26	2.82	35.44	3.57	9.93	394	536	70	1.58	40	2	0	Stagnosol
HEG014_11	6.28	39.21	0.33	38.88	3.61	10.75	450	491	60	2	19.8	2	116.76	Stagnosol
HEG015_11	7.1	55.11	1.79	53.32	5.24	10.16	528	435	38	1.82	38.7	1	75	Stagnosol
HEG016_11	6.61	64.89	0.85	64.04	6.39	10.02	555	405	39	1.24	164	0	0	Stagnosol
HEG017_11	6.99	54.11	0.94	53.17	4.96	10.71	546	419	32	0.77	63.9	0	0	Stagnosol
HEG018_11	7.32	66.61	18.47	48.14	4.1	11.74	457	446	97	0.81	70.6	0	0	Vertisol
HEG019_11	6.59	62.68	0.5	62.18	5.71	10.89	485	427	87	0.77	63.9	0	0	Stagnosol
HEG020_11	5.4	27.23	0	27.23	2.34	11.66	239	661	102	0.69	51.4	0	0	Stagnosol
HEG021_11	7.33	42.07	10.33	31.73	3.02	10.5	311	631	58	0.61	39.6	0	0	Stagnosol
HEG022_11	6.92	50.12	2.24	47.88	4.94	9.7	446	467	87	1.52	18.7	2	0	Cambisol
HEG023_11	7.2	50.17	3.09	47.08	4.75	9.9	588	375	37	1.28	62.3	1	0	Stagnosol
HEG024_11	6.63	48.98	0.81	48.17	5.08	9.48	545	404	52	1.45	110	1	0	Stagnosol
HEG025_11	7.26	59.54	4.34	55.2	5.6	9.87	423	531	46	1.77	334	0	0	Cambisol
HEG026_11	7.29	56.14	8.66	47.48	4.7	10.1	481	458	61	1.46	0	2	0	Cambisol
HEG027_11	7.29	48.76	4.28	44.49	4.26	10.43	492	472	36	1.6	0	1	60	Cambisol
HEG028_11	7.22	38.19	6.12	32.08	3.26	9.85	536	409	55	1.76	0	1	81	Cambisol
HEG029_11	7.12	30.56	1.02	29.54	2.87	10.28	469	501	30	1.76	0	1	81	Cambisol
HEG030_11	7.11	40.15	4.64	35.51	3.67	9.67	400	548	52	2.22	0	3	70	Cambisol
HEG031_11	7.15	46.29	5.26	41.03	4.09	10.02	237	726	36	2.25	104	1	121	Cambisol
HEG032_11	5.53	40.19	0.22	39.97	3.79	10.54	340	640	17	2.01	158	1	60	Cambisol
HEG033_11	5.02	40.08	0	40.08	3.84	10.45	353	618	29	2.1	140	1	81	Cambisol
HEG034_11	7.06	35.28	1.33	33.95	3.4	9.97	448	469	83	2.32	107	1	132	Cambisol
HEG035_11	6.97	50.8	5.05	45.74	4.79	9.56	307	580	113	2.32	107	1	132	Cambisol
HEG036_11	7.25	63.76	10.7	53.06	5.32	9.97	61	871	71	2.04	69.6	1	98	Cambisol
HEG037_11	7.41	66.94	14.32	52.61	5.39	9.75	60	841	99	2.1	69.6	1	108	Cambisol
HEG038_11	7.29	33.77	5.44	28.33	2.84	9.98	310	622	68	1.67	185	1	0	Cambisol
HEG039_11	6.46	38.87	0.43	38.44	3.9	9.85	351	537	110	1.24	165	0	0	Cambisol
HEG040_11	6.73	61.48	1.07	60.41	5.99	10.08	275	648	79	2.06	454	0	0	Cambisol
HEG041_11	7.21	41.98	2.17	39.81	3.52	11.29	518	417	65	0.69	51.4	0	0	Cambisol
HEG042_11	7.15	44	2.19	41.81	3.83	10.91	435	520	46	0.77	63.9	0	0	Cambisol
HEG043_11	7.16	46.85	1.44	45.41	4.07	11.16	376	589	38	1.42	101	1	0	Cambisol
HEG044_11	7.15	84.38	4.81	79.57	7.7	10.32	79	853	69	0.76	61.4	0	0	Cambisol
HEG045_11	6.73	44.99	0.55	44.44	4.27	10.39	492	468	43	0.85	76.8	0	0	Cambisol
HEG046_11	7.45	47.28	21.44	25.83	2.48	10.4	395	531	74	0.87	80.6	0	0	Cambisol
HEG047_11	7.09	58.25	3.16	55.09	5.57	9.89	483	477	40	1.86	141	2	0	Cambisol
HEG048_11	6.8	42.18	0.54	41.64	4.03	10.33	465	488	50	1.75	215	1	0	Cambisol
HEG049_11	6.63	47.17	0.67	46.5	4.65	10	431	500	71	1.51	16.3	2	0	Cambisol
HEG050_11	6.85	45.95	0.59	45.36	4.42	10.27	646	335	23	1.15	26.4	1	0	Cambisol

SEG001_11	7.24	218.5	14.93	203.5	19.1	10.65	233	466	301	3.1	0	3	50	Histosol
SEG002_11	7.32	171.2	42.76	128.4	12.7	10.1	198	527	275	2.82	88.9	1	50	Histosol
SEG003_11	7.42	135.5	45.7	89.82	8.89	10.1	89	600	311	2.92	0	2	50	Histosol
SEG004_11	7.35	210.5	69.4	141.1	13.9	10.15	163	686	151	1.03	0	1	0	Histosol
SEG005_11	7.42	181.2	66.56	114.7	11.51	9.96	162	575	263	1.46	0	2	0	Gleysol
SEG006_11	5.54	284.5	3.95	280.6	22.51	12.46	248	284	468	1.49	413	0	0	Histosol
SEG007_11	7.3	174.5	59.42	115	11.04	10.42	157	648	195	1.59	472	0	0	Histosol
SEG008_11	7.34	157.2	78.55	78.71	7.34	10.71	110	713	177	1.03	0	1	0	Gleysol
SEG009_11	6.56	233.6	4.11	229.4	18.2	12.6	171	143	686	0.94	163	0	0	Histosol
SEG010_11	7.4	205.4	58.16	147.3	14.21	10.36	183	516	301	2.92	0	2	50	Histosol
SEG011_11	7.43	183.8	71.32	112.5	11.48	9.8	137	552	311	2.92	0	2	50	Gleysol
SEG012_11	7.37	144.4	38.59	105.8	10.65	9.93	138	548	314	3.1	0	3	50	Histosol
SEG013_11	7.13	35.92	2.65	33.27	3.37	9.89	41	324	635	1.46	0	2	0	Cambisol
SEG014_11	7.35	172.3	68.55	103.8	10.45	9.94	169	625	206	0.82	124	0	0	Gleysol
SEG015_11	7.37	193.4	57.21	136.2	14.09	9.67	191	593	216	1.46	0	2	0	Histosol
SEG016_11	7.4	200.9	60.07	140.8	13.97	10.07	178	655	167	1.03	0	1	0	Gleysol
SEG017_11	5.03	355.8	4.11	351.7	27.77	12.66	374	348	278	1.15	243	0	0	Histosol
SEG018_11	5.14	14.87	0	14.87	1.21	12.3	65	89	846	1.46	0	2	0	Luvisol
SEG019_11	7.33	101.4	25.68	75.74	7.51	10.09	87	553	360	1.2	71	1	0	Gleysol
SEG020_11	6.59	276	6.49	269.5	21.54	12.51	307	304	389	1.4	165	1	0	Histosol
SEG021_11	5.35	283	2.71	280.3	21.84	12.83	278	310	412	1.21	271	0	0	Gleysol
SEG022_11	7.28	107.9	44.61	63.35	6.53	9.7	93	636	271	2.29	974	0	0	Gleysol
SEG023_11	5.2	296.6	3.68	292.9	20.71	14.14	373	461	166	2.5	0	2	32.3	Histosol
SEG024_11	7.07	232.1	28.81	203.3	20.16	10.08	283	504	213	1.03	0	1	0	Histosol
SEG025_11	6.31	202.3	4.56	197.8	16.21	12.19	176	206	618	1.46	0	2	0	Histosol
SEG026_11	7.14	305	7.94	297.1	24.17	12.29	427	353	220	2.5	0	2	32.3	Histosol
SEG027_11	5.76	370.8	11.29	359.5	24.69	14.56	416	417	167	1.03	0	1	0	Histosol
SEG028_11	7.3	202.3	56.98	145.3	13.63	10.66	175	651	174	1.03	0	1	0	Histosol
SEG029_11	7.39	181	49.45	131.6	12.94	10.16	195	694	110	1.03	0	1	0	Histosol
SEG030_11	7.09	31.96	2.56	29.4	2.79	10.52	146	343	511	1.46	0	2	0	Albeluvisol
SEG031_11	6.24	28.79	0.36	28.43	2.64	10.77	182	281	537	1.46	0	2	0	Cambisol
SEG032_11	5.91	24.18	0	24.18	2.3	10.5	190	234	576	1.46	0	2	0	Luvisol
SEG033_11	5.71	18.22	0	18.22	1.75	10.41	118	265	617	1.32	325	0	0	Albeluvisol
SEG034_11	5.58	18.41	0	18.41	1.74	10.59	138	273	589	1.58	151	0	85.14	Albeluvisol
SEG035_11	5.97	22.83	0	22.83	2.15	10.62	200	238	562	1.71	35.7	1	85.14	Luvisol
SEG036_11	6.2	24.58	0.29	24.28	2.41	10.09	139	301	560	1.36	344	0	0	Albeluvisol
SEG037_11	4.58	12.22	0	12.22	1.16	10.51	93	99	808	1.38	156	1	0	Albeluvisol
SEG038_11	5.17	22.82	0	22.82	2.18	10.46	89	72	838	1.57	259	1	0	Cambisol
SEG039_11	7.07	22.29	5.42	16.87	1.63	10.34	225	237	538	1.42	176	1	0	Cambisol
SEG040_11	5.76	21.3	0	21.3	2.03	10.49	98	192	710	1.74	560	0	0	Luvisol
SEG041_11	5.3	20.73	0	20.73	1.99	10.41	136	185	679	1.4	362	0	0	Luvisol
SEG042_11	5.08	18.64	0	18.64	1.85	10.06	117	289	594	2.08	801	0	0	Luvisol
SEG043_11	5.94	19.81	0.3	19.51	1.93	10.12	131	121	748	1.71	541	0	0	Luvisol
SEG044_11	5.41	21.83	0	21.83	2.15	10.15	113	189	698	0.76	107	0	0	Cambisol
SEG045_11	5.26	18.91	0	18.91	1.85	10.21	100	234	666	1.1	226	0	0	Albeluvisol
SEG046_11	5.37	24.3	0	24.3	2.3	10.58	146	210	644	1.77	582	0	0	Cambisol
SEG047_11	6.17	23.34	0.32	23.03	2.18	10.55	186	255	559	2.07	599	1	0	Luvisol
SEG048_11	6.73	18.16	0.62	17.54	1.73	10.11	130	120	750	1.57	460	0	0	Luvisol
SEG049_11	5.98	20.98	0	20.98	1.97	10.66	106	372	522	1.51	422	0	0	Albeluvisol
SEG050_11	5.07	23.05	0	23.05	2.13	10.84	92	112	796	1	187	0	0	Cambisol
AEG001_17	6.78	90.96	1.41	89.56	9.19	9.75	643	324	33	2.32	0	2	30	Leptosol
AEG002_17	6.86	82.5	2.22	80.27	8.42	9.53	512	411	77	13.54	0	3	433	Leptosol
AEG003_17	6.1	63.93	0	63.93	5.98	10.69	672	298	30	2.18	23.7	2	12.92	Leptosol
AEG004_17	5.27	51.1	0	51.1	5.26	9.71	488	427	85	2.52	75	1	30	Leptosol
AEG005_17	6.26	95.35	0.68	94.67	9.39	10.08	365	518	117	1.55	35.9	0	41.65	Leptosol
AEG006_17	6.02	84.56	0.4	84.17	8.38	10.05	588	380	32	4.99	245	1	40	Leptosol

AEG007_17	7.34	92.51	33.91	58.61	4.98	11.78	385	427	188	0.74	57.2	0	0	Leptosol
AEG008_17	6.61	91.86	0.46	91.4	8.39	10.89	673	319	8	1.39	47.7	1	0	Leptosol
AEG009_17	6.58	78.6	0.53	78.07	6.46	12.08	556	417	27	1.57	121	0	0	Leptosol
AEG010_17	5.85	94.09	0.44	93.65	9.14	10.24	647	327	26	1.02	19.4	1	0	Leptosol
AEG011_17	5.36	63.06	0	63.06	5.91	10.68	643	327	30	2.58	0	2	40	Leptosol
AEG012_17	6.63	74.58	1.52	73.06	7.11	10.28	436	512	52	2.32	0	2	30	Leptosol
AEG013_17	6.26	76.38	0.57	75.81	7.14	10.61	643	311	46	3.06	0	2	58.5	Leptosol
AEG014_17	6.6	64.81	0.65	64.16	6.36	10.09	521	453	26	3.09	0	2	60	Leptosol
AEG015_17	5.66	62.61	0	62.61	6.27	9.98	642	340	18	11.7	0	3	362	Leptosol
AEG016_17	5.97	72.88	0.36	72.52	7.08	10.24	528	449	23	2.83	159	1	0	Leptosol
AEG017_17	6.89	65.39	1.82	63.56	6.27	10.14	618	365	17	1.88	0	2	13.28	Leptosol
AEG018_17	6.94	72.9	3.75	69.15	7.01	9.87	649	332	19	6.24	0	3	151.6	Leptosol
AEG019_17	5.76	79.03	0.41	78.62	7.94	9.91	637	327	36	7	480	1	0	Leptosol
AEG020_17	6.69	89.97	0.84	89.13	9.42	9.47	597	334	69	2.23	172	0	0	Leptosol
AEG021_17	5.82	70.28	0.33	69.95	6.97	10.04	587	391	22	7.23	445	1	26.4	Leptosol
AEG022_17	5.68	56.61	0.35	56.27	5.05	11.14	708	270	22	1.09	24.8	1	0	Leptosol
AEG023_17	7.05	84.93	2.82	82.11	8.44	9.73	566	387	47	1.87	0	2	12.92	Leptosol
AEG024_17	6.08	80.65	0.51	80.14	7.94	10.1	638	329	33	5.42	33	3	103.5	Leptosol
AEG025_17	7.19	99.43	16.9	82.52	7.37	11.2	463	406	131	0.93	71.3	0	0	Leptosol
AEG026_17	6.84	74.78	1.01	73.77	6.12	12.06	679	241	80	3.46	267	0	0	Leptosol
AEG027_17	5.97	100.86	0.47	100.39	8.72	11.51	576	410	14	1.42	110	0	0	Leptosol
AEG028_17	6.13	60.01	0.33	59.69	5.21	11.46	599	385	16	1.11	85.9	0	0	Leptosol
AEG029_17	5.87	58.51	0.44	58.07	5.58	10.4	566	343	91	1.53	58.7	1	0	Cambisol
AEG030_17	6.64	64.4	0.56	63.85	6.47	9.86	556	375	69	1.18	32	1	0	Leptosol
AEG031_17	6.66	73.28	0.68	72.6	7.25	10.01	595	381	24	4.59	294	1	0	Leptosol
AEG032_17	5.41	46.34	0.3	46.04	4.12	11.16	461	466	73	0.78	59.8	0	0	Leptosol
AEG033_17	6	50.42	0.35	50.08	4.65	10.76	597	388	15	0.68	52.1	0	0	Leptosol
AEG034_17	6.33	47.3	0.43	46.87	4.72	9.93	423	554	23	1.66	68.8	1	0	Leptosol
AEG035_17	5.34	45.41	0.46	44.96	4.47	10.06	446	503	51	2.58	0	2	40	Cambisol
AEG036_17	5.97	59.44	0.5	58.94	5.58	10.57	588	213	199	3.72	0	2	84	Cambisol
AEG037_17	6.31	73.35	0.81	72.54	6.92	10.49	566	334	100	2.49	0	2	36.6	Cambisol
AEG038_17	5.62	53.76	0.29	53.47	5.51	9.7	451	468	81	1.96	0	2	16.15	Cambisol
AEG039_17	5.97	73.01	0.49	72.52	6.89	10.53	463	493	44	2.32	0	2	30	Cambisol
AEG040_17	6.86	73.61	1.7	71.91	7.36	9.76	698	269	33	4.66	0	3	90.73	Cambisol
AEG041_17	6.26	52.36	0.31	52.05	4.97	10.48	541	407	52	5.35	0	2	146.9	Cambisol
AEG042_17	7.12	99.44	16.14	83.3	8.84	9.42	159	757	84	3.09	0	3	30	Cambisol
AEG043_17	6.86	90.09	4.81	85.28	8.87	9.61	613	349	38	1.7	71.7	1	0	Cambisol
AEG044_17	7.27	85.85	14.18	71.67	7.49	9.56	152	735	113	2.97	229	0	0	Cambisol
AEG045_17	5.39	59.82	0.32	59.5	6.1	9.75	529	417	54	2.41	0	3	3.96	Cambisol
AEG046_17	6.04	80.61	0.49	80.11	8.21	9.76	659	287	54	3.58	276	0	0	Cambisol
AEG047_17	7.51	106.49	44.73	61.75	5.65	10.92	146	704	150	1.25	96.4	0	0	Cambisol
AEG048_17	7.56	97.63	55.36	42.27	3.47	12.18	295	565	140	0.61	47.3	0	0	Cambisol
AEG049_17	6.03	56.03	0.43	55.6	5.25	10.59	451	522	27	1.21	93.2	0	0	Cambisol
AEG050_17	6.04	75.07	0.48	74.59	7.41	10.06	589	382	29	2.39	0	3	3.3	Cambisol
HEG001_17	6.62	53.65	0.65	53	5.25	10.09	502	447	50	10.12	112	2	215.89	Cambisol
HEG002_17	7.26	42.16	4.97	37.19	3.88	9.59	552	396	52	5.95	0	2	130	Vertisol
HEG003_17	7.32	45.98	7.26	38.72	4.01	9.65	544	399	57	6.83	0	3	130	Vertisol
HEG004_17	6.54	66.24	0.55	65.69	6.16	10.67	511	414	75	5.11	0	2	104	Stagnosol
HEG005_17	7.16	46.84	4.86	41.98	4.33	9.69	454	497	49	5.25	0	3	81	Stagnosol
HEG006_17	5.85	24.42	0.28	24.14	2.39	10.11	257	698	45	5.33	14.4	2	105.11	Stagnosol
HEG007_17	7	57.5	4.29	53.21	5.6	9.5	536	434	30	2.58	208	0	0	Stagnosol
HEG008_17	7.01	70.05	3.06	67	6.55	10.22	494	452	56	2.58	208	0	0	Stagnosol
HEG009_17	7.1	45.93	2.23	43.7	4.15	10.53	387	537	76	1.62	59.8	1	0	Stagnosol
HEG010_17	6.54	51.95	0.44	51.51	4.56	11.29	436	532	30	1.65	24.9	1	14.25	Vertisol
HEG011_17	7.28	66.85	9.92	56.92	5.41	10.52	552	405	43	2.11	62.3	1	14.25	Stagnosol
HEG012_17	7.02	75.97	3.14	72.83	7.39	9.86	497	448	55	8.9	517	1	50	Stagnosol

HEG013_17	7.21	48.05	4.07	43.98	4.54	9.68	394	536	70	5.27	48	3	63.3	Stagnosol
HEG014_17	6.41	44.49	0.39	44.1	4.1	10.75	450	491	60	2.81	0	1	60	Stagnosol
HEG015_17	7.07	64.42	1.77	62.66	6.03	10.38	528	435	38	4.34	60.1	1	84	Stagnosol
HEG016_17	6.76	73.31	0.83	72.48	7.07	10.24	555	405	39	1.39	112	0	0	Stagnosol
HEG017_17	6.93	57.03	0.62	56.41	5.18	10.9	546	419	32	0.77	61.7	0	0	Stagnosol
HEG018_17	7.35	65.16	11.07	54.09	4.82	11.23	457	446	97	1.19	25	1	0	Vertisol
HEG019_17	6.6	64.67	0.47	64.2	5.81	11.05	485	427	87	0.77	61.7	0	0	Stagnosol
HEG020_17	5.52	29.73	0	29.73	2.64	11.27	239	661	102	1.62	59.8	1	0	Stagnosol
HEG021_17	7.3	46.68	9.42	37.25	3.6	10.35	311	631	58	2.01	91.6	1	0	Stagnosol
HEG022_17	6.88	53.2	1.81	51.39	5.23	9.83	446	467	87	2.97	97.5	2	0	Cambisol
HEG023_17	7.28	51.05	2.4	48.64	4.85	10.03	588	375	37	0.88	0	1	0	Stagnosol
HEG024_17	6.77	61.4	1.08	60.32	6.08	9.92	545	404	52	1.55	54.5	1	0	Stagnosol
HEG025_17	7.28	62.34	3.84	58.5	5.75	10.18	423	531	46	1.38	40.7	1	0	Cambisol
HEG026_17	7.37	57.04	9.92	47.12	4.85	9.72	481	458	61	0.88	0	1	0	Cambisol
HEG027_17	7.24	55.65	4.18	51.48	4.93	10.44	492	472	36	3.48	53.9	1	60	Cambisol
HEG028_17	7.32	45.17	3.63	41.54	4.21	9.87	536	409	55	1.72	0	1	26	Cambisol
HEG029_17	7.16	38.63	1.01	37.62	3.6	10.46	469	501	30	3	32.8	2	26	Cambisol
HEG030_17	7.19	49.27	3.07	46.21	4.77	9.68	400	548	52	5.69	61.9	3	71	Cambisol
HEG031_17	7.21	53.02	6.61	46.41	4.71	9.86	237	726	36	2.99	50.3	1	46	Cambisol
HEG032_17	5.61	41.82	0.29	41.53	3.93	10.57	340	640	17	3.78	77.9	1	60	Cambisol
HEG033_17	5.34	42.08	0	42.08	3.87	10.88	353	618	29	2.84	87.5	2	0	Cambisol
HEG034_17	6.95	40.85	1.26	39.59	4.01	9.87	448	469	83	5.33	14.4	2	105.11	Cambisol
HEG035_17	7.02	52.99	5.33	47.65	5	9.54	307	580	113	4.17	265	1	0	Cambisol
HEG036_17	7.27	71.26	12.17	59.09	5.93	9.97	61	871	71	1.56	115	0	4.28	Cambisol
HEG037_17	7.3	75.89	16.62	59.27	6.18	9.58	60	841	99	5.04	46.1	2	84	Cambisol
HEG038_17	7.32	36.49	5.35	31.15	3.28	9.49	310	622	68	4.17	336	0	0	Cambisol
HEG039_17	6.48	43.38	0.48	42.9	4.24	10.12	351	537	110	1.51	51.1	1	0	Cambisol
HEG040_17	6.56	68.7	1.09	67.61	6.69	10.11	275	648	79	4.58	369	0	0	Cambisol
HEG041_17	7.18	45.02	1.29	43.73	3.85	11.35	518	417	65	1.62	59.8	1	0	Cambisol
HEG042_17	7.24	48.67	3.85	44.82	4.2	10.68	435	520	46	0.77	61.7	0	0	Cambisol
HEG043_17	7.14	48.89	1.23	47.66	4.27	11.16	376	589	38	0.57	45.6	0	0	Cambisol
HEG044_17	7.14	87.53	4.83	82.7	8.33	9.93	79	853	69	0.66	53.3	0	0	Cambisol
HEG045_17	6.98	47.47	0.64	46.83	4.49	10.42	492	468	43	1.45	46.1	1	0	Cambisol
HEG046_17	7.42	51.11	24.05	27.06	2.96	9.13	395	531	74	1.72	68.1	1	0	Cambisol
HEG047_17	7.15	62.51	2.59	59.92	5.98	10.02	483	477	40	2.13	101	1	0	Cambisol
HEG048_17	7.02	48.19	0.8	47.39	4.55	10.41	465	488	50	1.43	44.3	1	0	Cambisol
HEG049_17	6.67	55.09	0.81	54.27	5.33	10.18	431	500	71	2.21	0	2	14.25	Cambisol
HEG050_17	6.89	50.6	1.02	49.58	4.71	10.52	646	335	23	1.75	70.6	1	0	Cambisol
SEG001_17	7.51	190.89	17.28	173.6	20.48	8.48	233	466	301	2.64	24.8	2	0	Histosol
SEG002_17	7.47	148.59	43.96	104.63	13.63	7.68	198	527	275	2.09	149	1	0	Histosol
SEG003_17	7.6	115.62	47.34	68.29	9.29	7.35	89	600	311	1.9	116	1	0	Histosol
SEG004_17	7.5	197.84	73.39	124.45	16.32	7.63	163	686	151	0	0	0	0	Histosol
SEG005_17	7.59	165.05	70.18	94.87	12.84	7.39	162	575	263	1.25	0	1	0	Gleysol
SEG006_17	5.49	248.12	3.44	244.68	25.41	9.63	248	284	468	1.33	235	0	0	Histosol
SEG007_17	7.48	147.4	59.9	87.5	11.58	7.55	157	648	195	2.52	225	1	0	Histosol
SEG008_17	7.47	138.22	79.56	58.65	8.14	7.21	110	713	177	4.3	319	2	0	Gleysol
SEG009_17	6.63	174.98	3.92	171.06	17.1	10	171	143	686	2.65	469	0	0	Histosol
SEG010_17	7.51	220.87	59.4	161.47	17.69	9.13	183	516	301	1.85	105	1	0	Histosol
SEG011_17	7.54	191.72	73.26	118.45	14.87	7.97	137	552	311	1.85	105	1	0	Gleysol
SEG012_17	7.45	135.33	40.2	95.13	12.57	7.57	138	548	314	2.64	24.8	2	0	Histosol
SEG013_17	5.54	21.76	0	21.76	2.4	9.08	41	324	635	25.71	0	2	10.26	Cambisol
SEG014_17	7.51	162.15	71.18	90.97	12.11	7.51	169	625	206	2.55	9.51	2	0	Gleysol
SEG015_17	7.46	167.96	59.69	108.26	14.6	7.41	191	593	216	1.3	8.9	1	0	Histosol
SEG016_17	7.43	179.48	63.07	116.42	15.34	7.59	178	655	167	0.14	24.1	0	0	Gleysol
SEG017_17	5.41	320.86	4.28	316.59	30.58	10.35	374	348	278	2.62	243	1	0	Histosol
SEG018_17	4.86	10.76	0	10.76	1.11	9.72	65	89	846	1.25	0	1	0	Luvisol

SEG019_17	7.53	92.35	25.06	67.29	8.55	7.87	87	553	360	1.67	74.7	1	0	Gleysol
SEG020_17	6.38	246.8	6.22	240.58	24.13	9.97	307	304	389	2.27	402	0	0	Histosol
SEG021_17	5.26	233.04	2.48	230.56	21.72	10.61	278	310	412	2.51	445	0	0	Gleysol
SEG022_17	7.48	95	47	48	6.82	7.04	93	636	271	0.36	64	0	0	Gleysol
SEG023_17	5.16	288.03	3.61	284.42	25.05	11.35	373	461	166	2.5	0	2	0	Histosol
SEG024_17	7.49	221.31	35.94	185.37	23.21	7.99	283	504	213	1.32	11.7	1	0	Histosol
SEG025_17	6.54	183.95	4.29	179.66	17.83	10.07	176	206	618	2.5	0	2	0	Histosol
SEG026_17	7.23	277.07	8.45	268.62	27.08	9.92	427	353	220	2.5	0	2	0	Histosol
SEG027_17	6.13	372.51	12.45	360.06	30.64	11.75	416	417	167	1.25	0	1	0	Histosol
SEG028_17	7.52	189.8	57.87	131.93	16.09	8.2	175	651	174	1.31	11.2	1	0	Histosol
SEG029_17	7.61	163.06	53.21	109.85	14.39	7.63	195	694	110	0.5	87.7	0	0	Histosol
SEG030_17	7.04	29.35	1.77	27.59	3.17	8.71	146	343	511	1.25	0	1	0	Albeluvisol
SEG031_17	5.95	27.5	0	27.5	2.69	10.21	182	281	537	1.25	0	1	0	Cambisol
SEG032_17	5.65	22.55	0	22.55	2.21	10.21	190	234	576	1.25	0	1	0	Luvisol
SEG033_17	5.69	20.09	0	20.09	1.98	10.15	118	265	617	1.67	295	0	0	Albeluvisol
SEG034_17	5.76	20.45	0	20.45	1.92	10.67	138	273	589	2.34	192	1	0	Albeluvisol
SEG035_17	6.18	25.23	0	25.23	2.43	10.37	200	238	562	0.97	171	0	0	Luvisol
SEG036_17	6.29	24.61	0	24.61	2.36	10.42	139	301	560	2.1	151	1	0	Albeluvisol
SEG037_17	4.71	14.37	0	14.37	1.31	10.95	93	99	808	0.45	79.9	0	0	Albeluvisol
SEG038_17	5.15	18.47	0	18.47	1.71	10.8	89	72	838	6.48	925	1	0	Cambisol
SEG039_17	7.4	25.96	6.22	19.74	1.95	10.13	225	237	538	2.89	68.8	2	0	Cambisol
SEG040_17	6.12	45.64	0	45.64	4.39	10.4	98	192	710	1.05	186	0	0	Luvisol
SEG041_17	6.11	24.51	0	24.51	2.37	10.34	136	185	679	3.11	330	1	0	Luvisol
SEG042_17	5.01	21.09	0	21.09	2.08	10.15	117	289	594	2.99	528	0	0	Luvisol
SEG043_17	6.51	29.27	0.36	28.91	2.77	10.42	131	121	748	28.14	239	0	11.84	Luvisol
SEG044_17	5.45	21.63	0	21.63	2.1	10.3	113	189	698	0.96	169	0	0	Cambisol
SEG045_17	5.89	18.83	0	18.83	1.79	10.51	100	234	666	1.01	179	0	0	Albeluvisol
SEG046_17	7.07	38.57	4.1	34.48	3.59	9.61	146	210	644	5.07	676	1	0	Cambisol
SEG047_17	5.7	26.15	0	26.15	2.37	11.02	186	255	559	2.5	221	1	0	Luvisol
SEG048_17	6.67	15.81	0.34	15.47	1.56	9.89	130	120	750	2.95	521	0	0	Luvisol
SEG049_17	6.36	22.33	0.37	21.96	2.15	10.22	106	372	522	2.14	378	0	0	Albeluvisol
SEG050_17	5.44	24.83	0	24.83	2.19	11.36	92	112	796	2.15	380	0	0	Cambisol

1: (cattle*day)/year

2: (cuts/year)

10580
Grassland_management
meadow
meadow
meadow
mowed_meadow
mowed_meadow
mowed_meadow
pasture
pasture
pasture
meadow
meadow
meadow
meadow
meadow
meadow
mowed_meadow
meadow
meadow
mowed_meadow
pasture
pasture
meadow
meadow
mowed_meadow
pasture
pasture
pasture
pasture
mowed_meadow
mowed_meadow
mowed_meadow
pasture
pasture
pasture
meadow
meadow
meadow
meadow
meadow
meadow
meadow
mowed_meadow
mowed_meadow
pasture

meadow
pasture
pasture
pasture
pasture
meadow
meadow
meadow
meadow
mowed_meadow
mowed_meadow
mowed_meadow
pasture
pasture
pasture
meadow
meadow
mowed_meadow
mowed_meadow
mowed_meadow
mowed_meadow
pasture
pasture
pasture
pasture
pasture
pasture
mowed_meadow
mowed_meadow
mowed_meadow
pasture
meadow
meadow
mowed_meadow
mowed_meadow
mowed_meadow
mowed_meadow
mowed_meadow
pasture
mowed_meadow
mowed_meadow
mowed_meadow
mowed_meadow
pasture
pasture
pasture
pasture
pasture
pasture
pasture
pasture
pasture
mowed_meadow
mowed_meadow
mowed_meadow
mowed_meadow

meadow
meadow
meadow
mowed_meadow
mowed_meadow
mowed_meadow
pasture
pasture
pasture
meadow
meadow
meadow
meadow
pasture
mowed_meadow
mowed_meadow
mowed_meadow
meadow
pasture
pasture
pasture
pasture
meadow
meadow
meadow
meadow
meadow
meadow
meadow
meadow
meadow
meadow
mowed_meadow
mowed_meadow
mowed_meadow
pasture
pasture
mowed_meadow
pasture
pasture
pasture
pasture
pasture
pasture
pasture
pasture
pasture
pasture
pasture
pasture
meadow
meadow
meadow
mowed_meadow
mowed_meadow
mowed_meadow

pasture
pasture
pasture
meadow
meadow
meadow
meadow
meadow
meadow
mowed_meadow
meadow
meadow
mowed_meadow
pasture
pasture
meadow
meadow
mowed_meadow
pasture
pasture
pasture
pasture
mowed_meadow
mowed_meadow
mowed_meadow
pasture
pasture
pasture
meadow
meadow
meadow
meadow
meadow
meadow
meadow
mowed_meadow
mowed_meadow
pasture
meadow
pasture
pasture
pasture
pasture
meadow
meadow
meadow
meadow
mowed_meadow
mowed_meadow
mowed_meadow
pasture
pasture
pasture
meadow
meadow
mowed_meadow

mowed_meadow
mowed_meadow
mowed_meadow
pasture
pasture
pasture
pasture
pasture
pasture
mowed_meadow
mowed_meadow
mowed_meadow
pasture
meadow
meadow
mowed_meadow
mowed_meadow
mowed_meadow
mowed_meadow
mowed_meadow
pasture
mowed_meadow
mowed_meadow
mowed_meadow
mowed_meadow
pasture
pasture
pasture
pasture
pasture
pasture
pasture
pasture
pasture
mowed_meadow
mowed_meadow
mowed_meadow
mowed_meadow
meadow
meadow
meadow
mowed_meadow
mowed_meadow
mowed_meadow
pasture
pasture
pasture
meadow
meadow
meadow
meadow
pasture
mowed_meadow
mowed_meadow
mowed_meadow
meadow

pasture
pasture
pasture
pasture
meadow
meadow
meadow
meadow
meadow
meadow
meadow
meadow
meadow
meadow
mowed_meadow
mowed_meadow
mowed_meadow
pasture
pasture
mowed_meadow
pasture
pasture
pasture
pasture
pasture
pasture
pasture
pasture
pasture
pasture
pasture
pasture
