# Supplementary material for: Contrasting Responses of Protistan Plant Parasites and Phagotrophs to Ecosystems, Land Management and Soil Properties
Source: Front Microbiol. 2020 Aug 5;11:1823. doi: 10.3389/fmicb.2020.01823 (PMC7422690; doi:10.3389/fmicb.2020.01823)
Supplement: Supplementary file 3 [file Data_Sheet_3.zip › Table S2.pdf]

Table S2. Environmental parameters from the 150 forest study sites and two years of collection.

Reference dataset at https://www.bexis.uni-jena.de/	14447 22246	14446 23846	14446 23846	14446 23846	14446 23846	14446 23846	14686			16466	10580	17706	17706
Sites (AE=Alb, HE=Hainich, SE=Schorfheide, 11/17:year of collection	pH	Total_C g/kg soil	Inorganic_C g/kg	Organic_ C g/kg	Total_N g/kg	CN_ratio	Clay	Silt	Sand	Intensity_management[1]	soil_type	main_tree_ species	Developmental_ stage[2]
AEF001_11	3.43	56.53	0.24	56.29	3.67	15.34	318	659	23	1.8052	Cambisol	Pa	30_50
AEF001_17	3.34	72.74	0.34	72.41	4.47	16.2	318	659	23	1.8052	Cambisol	Pa	30_50
AEF002_11	4.34	53.26	0.23	53.03	3.81	13.91	500	462	38	2.2656	Leptosol	Pa	30_50
AEF002_17	4.84	53.2	0.32	52.88	3.82	13.85	500	462	38	2.2656	Leptosol	Pa	30_50
AEF003_11	5.66	69.24	0.72	68.52	4.83	14.18	527	424	49	2.3526	Cambisol	Pa	30_50
AEF003_17	5.63	64.02	0.56	63.46	4.67	13.58	527	424	49	2.3526	Cambisol	Pa	30_50
AEF004_11	6.2	71.22	0.9	70.32	5.51	12.75	487	482	32	1.5633	Cambisol	Fs	15_30
AEF004_17	6.76	77.36	3.85	73.51	6	12.25	487	482	32	1.5633	Cambisol	Fs	15_30
AEF005_11	4.51	48.61	0.21	48.4	3.73	12.98	404	579	18	0.9392	Cambisol	Fs	50_100
AEF005_17	4.45	46.44	0.33	46.11	3.58	12.86	404	579	18	0.9392	Cambisol	Fs	50_100
AEF006_11	4.9	39.78	0.19	39.58	3.21	12.32	415	494	91	1.1882	Cambisol	Fs	30_50
AEF006_17	5.59	47.95	0.44	47.51	3.76	12.63	415	494	91	1.1882	Cambisol	Fs	30_50
AEF007_11	4.6	62.64	0.22	62.42	4.78	13.05	548	362	90	1.0285	Leptosol	Fs	50_100
AEF007_17	5.01	62.89	0.39	62.5	4.85	12.89	548	362	90	1.0285	Leptosol	Fs	50_100
AEF008_11	6.08	62.18	1.77	60.41	4.28	14.11	492	466	42	0	Cambisol	Fs	50_100
AEF008_17	6.43	60.06	1.17	58.89	4.17	14.11	492	466	42	0	Cambisol	Fs	50_100
AEF009_11	5.9	58.64	0.83	57.81	4.05	14.29	693	289	18	0.4853	Leptosol	Fs	50_100
AEF009_17	6.12	61.25	0.66	60.59	4.27	14.2	693	289	18	0.4853	Leptosol	Fs	50_100
AEF010_11	4.63	57.48	0.24	57.24	4.24	13.48	528	441	33	2.1337	Leptosol	Pa	30_50
AEF010_17	4.63	47.34	0.33	47.02	3.77	12.46	528	441	33	2.1337	Leptosol	Pa	30_50
AEF011_11	3.49	54.13	0.24	53.89	3.57	15.09	260	699	41	1.6501	Cambisol	Pa	50_100
AEF011_17	3.42	60.03	0.3	59.73	3.92	15.22	260	699	41	1.6501	Cambisol	Pa	50_100
AEF012_11	4.29	60.44	0.29	60.16	4.57	13.16	570	405	25	2.3458	Cambisol	Pa	50_100
AEF012_17	4.52	58.27	0.37	57.9	4.49	12.89	570	405	25	2.3458	Cambisol	Pa	50_100
AEF013_11	4.76	63.93	0.27	63.67	4.29	14.85	666	289	45	1.7833	Cambisol	Pa	50_100
AEF013_17	5.16	59.49	0.31	59.18	3.99	14.82	666	289	45	1.7833	Cambisol	Pa	50_100
AEF014_11	4.59	42.17	0.27	41.9	3.27	12.81	449	508	43	1.7546	Cambisol	Pa	50_100
AEF014_17	4.84	46.57	0	46.57	3.49	13.34	449	508	43	1.7546	Cambisol	Pa	50_100
AEF015_11	6.23	94.53	0.98	93.55	6.8	13.76	653	277	70	2.822	Leptosol	Fs	0-15
AEF015_17	6.37	65.02	1.01	64.01	4.8	13.34	653	277	70	2.822	Leptosol	Fs	0-15
AEF016_11	6.2	60.63	0.67	59.96	4.34	13.81	521	335	144	1.0864	Cambisol	Fs	0-15
AEF016_17	6.37	67.5	0.9	66.61	4.9	13.6	521	335	144	1.0864	Cambisol	Fs	0-15
AEF017_11	6.44	53.26	1.55	51.72	4.13	12.5	428	540	32	1.2564	Cambisol	Fs	15_30
AEF017_17	6.53	52.02	1.1	50.92	4.14	12.31	428	540	32	1.2564	Cambisol	Fs	15_30
AEF018_11	4.62	35.96	0.22	35.73	2.81	12.71	313	655	34	0.3935	Cambisol	Fs	50_100
AEF018_17	4.69	43.55	0.29	43.25	3.36	12.88	313	655	34	0.3935	Cambisol	Fs	50_100
AEF019_11	4.8	60.17	0.28	59.89	4.96	12.08	471	503	28	0.6589	Cambisol	Fs	50_100
AEF019_17	5.07	58.13	0.47	57.66	4.74	12.16	471	503	28	0.6589	Cambisol	Fs	50_100
AEF020_11	6.64	59.6	1.28	58.32	4.8	12.14	437	533	30	1.0784	Cambisol	Fs	50_100
AEF020_17	6.6	72.16	1.36	70.8	6.08	11.65	437	533	30	1.0784	Cambisol	Fs	50_100
AEF021_11	6.11	72.49	0.62	71.87	6.14	11.71	519	453	30	1.0136	Cambisol	Fs	50_100
AEF021_17	6.31	71.96	0.81	71.15	6.08	11.7	519	453	30	1.0136	Cambisol	Fs	50_100
AEF022_11	6.25	59.29	0.56	58.73	4.41	13.31	542	409	49	0.7178	Cambisol	Fs	30_50
AEF022_17	6.31	58.86	0.71	58.15	4.53	12.84	542	409	49	0.7178	Cambisol	Fs	30_50
AEF023_11	5.48	58.19	0.38	57.81	4.65	12.42	500	486	15	1.2499	Cambisol	Fs	50_100

AEF023_17	5.59	56.94	0.38	56.55	4.51	12.53	500	486	15	1.2499	Cambisol	Fs	50_100
AEF024_11	4.88	57.54	0.3	57.25	4.39	13.04	510	307	183	2.5668	Cambisol	Fs	0-15
AEF024_17	5.29	58.61	0.3	58.31	4.55	12.8	510	307	183	2.5668	Cambisol	Fs	0-15
AEF025_11	4.7	39.19	0.24	38.95	3.02	12.9	383	506	111	1.7803	Cambisol	Fs	0-15
AEF025_17	5.13	41.73	0	41.73	3.26	12.79	383	506	111	1.7803	Cambisol	Fs	0-15
AEF026_11	4.85	47.45	0.22	47.24	3.87	12.19	480	329	181	1.6704	Cambisol	Fs	15_30
AEF026_17	5.1	52.96	0	52.96	4.09	12.95	480	329	181	1.6704	Cambisol	Fs	15_30
AEF027_11	4.49	58.5	0.41	58.09	4.21	13.8	518	352	130	2.6402	Leptosol	Fs	15_30
AEF027_17	4.58	50.21	0	50.21	3.72	13.5	518	352	130	2.6402	Leptosol	Fs	15_30
AEF028_11	4.9	61.57	0.29	61.28	4.8	12.75	474	474	52	1.2266	Cambisol	Fs	30_50
AEF028_17	4.73	54.18	0.31	53.87	4.12	13.06	474	474	52	1.2266	Cambisol	Fs	30_50
AEF029_11	4.49	51.6	0.24	51.36	3.69	13.92	413	550	37	2.0625	Leptosol	Fs	30_50
AEF029_17	4.4	53.18	0	53.18	3.79	14.02	413	550	37	2.0625	Leptosol	Fs	30_50
AEF030_11	5.94	86.82	0.89	85.92	6.84	12.55	700	263	37	1.1701	Cambisol	Fs	30_50
AEF030_17	5.82	84.03	0.91	83.12	6.6	12.59	700	263	37	1.1701	Cambisol	Fs	30_50
AEF031_11	5.81	70.31	0.59	69.72	5.71	12.2	548	431	21	2.2552	Leptosol	Pa	30_50
AEF031_17	5.59	61.18	0.44	60.74	4.97	12.21	548	431	21	2.2552	Leptosol	Pa	30_50
AEF032_11	6.58	105.7	6.68	99.06	7.64	12.95	395	548	57	2.3871	Leptosol	Pa	30_50
AEF032_17	6.93	114.43	11.03	103.4	7.72	13.4	395	548	57	2.3871	Leptosol	Pa	30_50
AEF033_11	5.33	87.84	0.52	87.32	6.06	14.4	681	305	14	2.305	Cambisol	Pa	30_50
AEF033_17	5.79	92.8	0.87	91.92	6.06	15.16	681	305	14	2.305	Cambisol	Pa	30_50
AEF034_11	4.93	70.84	0.32	70.51	5.15	13.7	626	353	23	2.2675	Leptosol	Pa	30_50
AEF034_17	4.9	72.42	0.38	72.04	5.15	14	626	353	23	2.2675	Leptosol	Pa	30_50
AEF035_11	5.11	83.17	0.42	82.75	5.77	14.34	587	362	51	2.6049	Leptosol	Fs	0-15
AEF035_17	5.48	79.91	0.48	79.43	5.61	14.15	587	362	51	2.6049	Leptosol	Fs	0-15
AEF036_11	5.85	65.2	0.56	64.63	5.36	12.06	407	541	52	1.9237	Leptosol	Fs	0-15
AEF036_17	5.98	62.41	0.49	61.92	4.82	12.86	407	541	52	1.9237	Leptosol	Fs	0-15
AEF037_11	5.4	59.22	0.38	58.85	4.73	12.44	553	374	73	0.9939	Leptosol	Fs	0-15
AEF037_17	5.22	58.76	0.35	58.41	4.58	12.75	553	374	73	0.9939	Leptosol	Fs	0-15
AEF038_11	6.17	56.64	0.63	56.01	4.17	13.42	502	387	111	1.5666	Leptosol	Fs	15_30
AEF038_17	6.88	57.3	1.49	55.81	4.06	13.75	502	387	111	1.5666	Leptosol	Fs	15_30
AEF039_11	5.75	59.61	0.53	59.07	4.51	13.1	432	530	38	1.2416	Leptosol	Fs	15_30
AEF039_17	5.17	53.14	0.39	52.75	3.95	13.36	432	530	38	1.2416	Leptosol	Fs	15_30
AEF040_11	5.01	75.7	0.33	75.36	6.58	11.46	610	347	44	0.9907	Leptosol	Fs	50_100
AEF040_17	5.32	81.85	0.41	81.43	6.67	12.22	610	347	44	0.9907	Leptosol	Fs	50_100
AEF041_11	5.12	60.31	0.41	59.91	4.87	12.3	475	449	76	1.0057	Leptosol	Fs	30_50
AEF041_17	5.67	61.93	0.49	61.43	4.77	12.88	475	449	76	1.0057	Leptosol	Fs	30_50
AEF042_11	5.88	65.27	0.55	64.72	5.15	12.57	504	386	110	1.2601	Leptosol	Fs	30_50
AEF042_17	6.45	72.1	0.86	71.24	5.54	12.87	504	386	110	1.2601	Leptosol	Fs	30_50
AEF043_11	4.8	37.01	0.22	36.79	3.32	11.06	386	577	39	1.0001	Leptosol	Fs	50_100
AEF043_17	5.07	42.94	0	42.94	3.23	13.28	386	577	39	1.0001	Leptosol	Fs	50_100
AEF044_11	5.89	61.59	0.4	61.19	4.97	12.31	429	528	43	0.7599	Leptosol	Fs	0-15
AEF044_17	6.04	63.6	0.45	63.16	4.76	13.27	429	528	43	0.7599	Leptosol	Fs	0-15
AEF045_11	5.71	63.39	0.42	62.96	5.15	12.22	220	574	206	1.0005	Cambisol	Fs	0-15
AEF045_17	5.8	64.12	0.36	63.76	5.15	12.37	220	574	206	1.0005	Cambisol	Fs	0-15
AEF046_11	5.07	53.51	0.28	53.22	4.14	12.85	546	425	27	1.1088	Leptosol	Fs	30_50
AEF046_17	5.45	55.54	0.31	55.22	4.1	13.46	546	425	27	1.1088	Leptosol	Fs	30_50
AEF047_11	5.07	67.28	0.39	66.88	5.21	12.84	531	404	65	1.1534	Cambisol	Fs	30_50
AEF047_17	5.15	60.63	0.29	60.33	4.63	13.03	531	404	65	1.1534	Cambisol	Fs	30_50
AEF048_11	5.4	59.08	0.41	58.67	4.82	12.18	464	508	28	1.5792	Leptosol	Fs	30_50
AEF048_17	5.75	69.75	0.48	69.28	5.25	13.2	464	508	28	1.5792	Leptosol	Fs	30_50
AEF049_11	5.86	75.33	0.66	74.67	5.9	12.65	645	234	121	0.7165	Leptosol	Fs	50_100
AEF049_17	6.31	77.28	0.68	76.6	5.79	13.23	645	234	121	0.7165	Leptosol	Fs	50_100
AEF050_11	5.87	100.4	0.9	99.55	7.55	13.19	543	429	28	0.9005	Leptosol	Fs	50_100

AEF050_17	5.88	99.55	0.97	98.58	7.21	13.68	543	429	28	0.9005	Leptosol	Fs	50_100
HEF001_11	6.78	49.15	3.58	45.57	3.63	12.54	195	738	71	1.8694	Stagnosol	Pa	50_100
HEF001_17	6.23	51.33	1.11	50.22	3.91	12.85	195	738	71	1.8694	Stagnosol	Pa	50_100
HEF002_11	5.53	41.63	0.39	41.24	2.71	15.23	308	600	92	1.3073	Stagnosol	Pa	30_50
HEF002_17	6.59	68.36	1.08	67.28	3.83	17.55	308	600	92	1.3073	Stagnosol	Pa	30_50
HEF003_11	4.98	43.33	0.33	43	2.72	15.79	409	549	43	2.1979	Luvisol	Pa	30_50
HEF003_17	5.07	59.14	0.47	58.68	3.56	16.47	409	549	43	2.1979	Luvisol	Pa	30_50
HEF004_11	6	47.07	0.46	46.61	3.68	12.66	387	562	55	1.8905	Luvisol	Fs	0-15
HEF004_17	6.14	63.48	0.53	62.95	4.77	13.19	387	562	55	1.8905	Luvisol	Fs	0-15
HEF005_11	4.99	43.84	0.61	43.22	3.42	12.64	460	485	59	0.9556	Luvisol	Fs	30_50
HEF005_17	5.32	52.23	0.79	51.45	3.94	13.05	460	485	59	0.9556	Luvisol	Fs	30_50
HEF006_11	4.15	22.88	0.22	22.66	1.82	12.46	218	709	74	0.7467	Luvisol	Fs	50_100
HEF006_17	4.35	31.98	0	31.98	2.5	12.79	218	709	74	0.7467	Luvisol	Fs	50_100
HEF007_11	4.1	28.98	0.22	28.76	2.06	13.95	197	714	88	0.6948	Luvisol	Fs	50_100
HEF007_17	4.14	32.6	0	32.6	2.37	13.75	197	714	88	0.6948	Luvisol	Fs	50_100
HEF008_11	5.56	27.87	0.28	27.59	1.98	13.93	227	716	58	1.1562	Luvisol	Fs	50_100
HEF008_17	5.68	35.1	0	35.1	2.58	13.62	227	716	58	1.1562	Luvisol	Fs	50_100
HEF009_11	4.12	30.24	0.28	29.97	2.34	12.78	287	622	92	1.2574	Luvisol	Fs	50_100
HEF009_17	4.38	29.2	0	29.2	2.15	13.56	287	622	92	1.2574	Luvisol	Fs	50_100
HEF010_11	4.86	50.88	0.33	50.55	4.04	12.5	447	503	53	0.0641	Stagnosol	Fs	50_100
HEF010_17	4.93	52.41	0	52.41	4.03	13	447	503	53	0.0641	Stagnosol	Fs	50_100
HEF011_11	4.53	37.62	0.3	37.32	3.02	12.35	414	551	36	0.5177	Luvisol	Fs	50_100
HEF011_17	4.87	50.69	0	50.69	3.83	13.22	414	551	36	0.5177	Luvisol	Fs	50_100
HEF012_11	4.05	20.93	0	20.93	1.56	13.41	168	713	117	0	Luvisol	Fs	50_100
HEF012_17	4.14	28.86	0	28.86	2.03	14.21	168	713	117	0	Luvisol	Fs	50_100
HEF013_11	6.66	70.65	1.4	69.25	4.58	15.13	510	444	48	2.2452	Luvisol	Pa	30_50
HEF013_17	6.76	78.11	1.54	76.58	4.83	15.84	510	444	48	2.2452	Luvisol	Pa	30_50
HEF014_11	4.54	44.47	0.39	44.08	3.34	13.21	448	504	47	1.6478	Luvisol	Fs	0-15
HEF014_17	5.05	60.73	0.49	60.23	4.35	13.86	448	504	47	1.6478	Luvisol	Fs	0-15
HEF015_11	3.91	20.86	0	20.86	1.29	16.2	150	785	66	1.8782	Luvisol	Fs	0-15
HEF015_17	3.99	28.14	0	28.14	1.89	14.89	150	785	66	1.8782	Luvisol	Fs	0-15
HEF016_11	4.74	33.26	0.26	33.01	2.73	12.1	322	628	53	0.793	Luvisol	Fs	15_30
HEF016_17	4.86	35.88	0	35.88	2.87	12.49	322	628	53	0.793	Luvisol	Fs	15_30
HEF017_11	3.86	27.28	0	27.28	1.79	15.24	183	725	96	1.1114	Luvisol	Fs	15_30
HEF017_17	3.87	38.17	0	38.17	2.55	14.96	183	725	96	1.1114	Luvisol	Fs	15_30
HEF018_11	4.79	35.14	0.23	34.91	2.69	12.99	272	684	41	1.3244	Stagnosol	Fs	15_30
HEF018_17	5.55	42.56	0.55	42.01	3.05	13.78	272	684	41	1.3244	Stagnosol	Fs	15_30
HEF019_11	4.57	31.94	0.28	31.66	2.78	11.4	310	636	52	1.0176	Luvisol	Fs	30_50
HEF019_17	4.61	33.75	0	33.75	2.98	11.33	310	636	52	1.0176	Luvisol	Fs	30_50
HEF020_11	5.97	32.59	0.65	31.94	2.46	12.97	253	708	40	0.9926	Luvisol	Fs	30_50
HEF020_17	6.68	45.16	3.28	41.88	3.25	12.89	253	708	40	0.9926	Luvisol	Fs	30_50
HEF021_11	4.71	29.44	0.31	29.13	2.34	12.46	268	672	61	0.8144	Luvisol	Fs	50_100
HEF021_17	6.29	41.04	1.64	39.39	2.95	13.35	268	672	61	0.8144	Luvisol	Fs	50_100
HEF022_11	4.63	23.33	0	23.33	1.67	13.94	184	747	68	0.6386	Luvisol	Fs	50_100
HEF022_17	4.82	27.92	0	27.92	2.01	13.87	184	747	68	0.6386	Luvisol	Fs	50_100
HEF023_11	4.53	34.49	0.22	34.27	2.73	12.56	279	662	56	1.3847	Luvisol	Fs	50_100
HEF023_17	4.72	41.31	0	41.31	3.18	12.99	279	662	56	1.3847	Luvisol	Fs	50_100
HEF024_11	4.05	22.31	0.44	21.87	1.59	13.73	162	793	44	0.8871	Luvisol	Fs	50_100
HEF024_17	3.98	24.67	0	24.67	1.8	13.74	162	793	44	0.8871	Luvisol	Fs	50_100
HEF025_11	4.58	39.61	0.25	39.35	3.09	12.73	325	624	53	1.2635	Luvisol	Fs	50_100
HEF025_17	4.74	43.96	0	43.96	3.43	12.8	325	624	53	1.2635	Luvisol	Fs	50_100
HEF026_11	4.46	24.42	0	24.42	1.63	14.95	150	796	54	1.1968	Luvisol	Fs	50_100
HEF026_17	4.31	25.49	0	25.49	1.79	14.26	150	796	54	1.1968	Luvisol	Fs	50_100
HEF027_11	6.37	50.1	0.68	49.42	3.82	12.93	291	643	67	0.7979	Luvisol	Fs	50_100

HEF027_17	6.02	56.31	0	56.31	4.2	13.4	291	643	67	0.7979	Luvisol	Fs	50_100
HEF028_11	6.29	44.68	0.53	44.15	3.2	13.8	306	634	60	1.4438	Stagnosol	Fs	50_100
HEF028_17	6.19	53.61	0.63	52.98	3.83	13.83	306	634	60	1.4438	Stagnosol	Fs	50_100
HEF029_11	3.86	25.9	0	25.9	1.86	13.94	227	719	55	0.5728	Luvisol	Fs	50_100
HEF029_17	4.12	33.64	0	33.64	2.48	13.58	227	719	55	0.5728	Luvisol	Fs	50_100
HEF030_11	3.86	33.78	0.29	33.48	2.66	12.57	358	595	48	0.7626	Luvisol	Fs	50_100
HEF030_17	4.06	37.21	0	37.21	2.94	12.64	358	595	48	0.7626	Luvisol	Fs	50_100
HEF031_11	3.88	33.36	0.32	33.04	2.62	12.62	377	573	52	1.0788	Luvisol	Fs	50_100
HEF031_17	4.13	42.14	0	42.14	3.22	13.08	377	573	52	1.0788	Luvisol	Fs	50_100
HEF032_11	3.92	36.27	0.25	36.02	2.81	12.84	345	593	64	1.15	Luvisol	Fs	50_100
HEF032_17	3.93	49.21	0	49.21	3.6	13.67	345	593	64	1.15	Luvisol	Fs	50_100
HEF033_11	4.45	27.93	0.23	27.7	1.95	14.17	227	706	68	1.1054	Luvisol	Fs	50_100
HEF033_17	4.8	39.09	0	39.09	2.5	15.63	227	706	68	1.1054	Luvisol	Fs	50_100
HEF034_11	4.62	38.01	0.25	37.75	3.19	11.84	380	571	52	0	Luvisol	Fs	50_100
HEF034_17	4.66	40.1	0	40.1	3.25	12.35	380	571	52	0	Luvisol	Fs	50_100
HEF035_11	4.5	36.45	0.27	36.18	2.91	12.44	396	551	55	0.861	Luvisol	Fs	50_100
HEF035_17	4.44	40.64	0	40.64	3.33	12.19	396	551	55	0.861	Luvisol	Fs	50_100
HEF036_11	4.56	37.76	0.24	37.52	2.92	12.84	356	558	84	0.3653	Luvisol	Fs	50_100
HEF036_17	4.71	44.54	0	44.54	3.25	13.71	356	558	84	0.3653	Luvisol	Fs	50_100
HEF037_11	4.5	31.8	0.23	31.57	2.5	12.61	265	661	78	0	Stagnosol	Fs	50_100
HEF037_17	4.41	36.75	0	36.75	2.67	13.74	265	661	78	0	Stagnosol	Fs	50_100
HEF038_11	5.15	53.04	0.37	52.67	4.04	13.04	511	440	54	0.048	Luvisol	Fs	50_100
HEF038_17	5.42	70.91	0.49	70.42	5.24	13.45	511	440	54	0.048	Luvisol	Fs	50_100
HEF039_11	4.14	31.88	0.25	31.63	2.5	12.64	330	615	58	0.0928	Luvisol	Fs	50_100
HEF039_17	4.52	38.87	0	38.87	3.14	12.37	330	615	58	0.0928	Luvisol	Fs	50_100
HEF040_11	5.47	48.95	0.44	48.51	4.02	12.05	406	563	33	0.023	Luvisol	Fs	50_100
HEF040_17	5.43	53.1	0	53.1	3.94	13.47	406	563	33	0.023	Luvisol	Fs	50_100
HEF041_11	4.26	24.25	0	24.25	1.94	12.52	210	754	34	0.32	Luvisol	Fs	50_100
HEF041_17	4.55	31.89	0	31.89	2.51	12.7	210	754	34	0.32	Luvisol	Fs	50_100
HEF042_11	3.97	23.41	0	23.41	1.63	14.34	184	760	60	0.54	Stagnosol	Fs	50_100
HEF042_17	4.17	31.24	0	31.24	2.14	14.62	184	760	60	0.54	Stagnosol	Fs	50_100
HEF043_11	6.35	50.98	1.04	49.94	3.89	12.83	337	635	30	1.8559	Stagnosol	Fs	0-15
HEF043_17	6.72	69.24	2.6	66.64	4.86	13.71	337	635	30	1.8559	Stagnosol	Fs	0-15
HEF044_11	6.27	46.69	0.8	45.89	3.42	13.42	373	586	43	1.7997	Stagnosol	Fs	0-15
HEF044_17	5.36	56.58	0.48	56.1	3.93	14.27	373	586	43	1.7997	Stagnosol	Fs	0-15
HEF045_11	7.1	67.36	9.94	57.42	4.6	12.5	88	854	60	1.0203	Luvisol	Fs	15_30
HEF045_17	7.15	78.62	9.26	69.36	5.18	13.39	88	854	60	1.0203	Luvisol	Fs	15_30
HEF046_11	3.92	37.14	0.22	36.92	2.91	12.7	387	570	45	1.0506	Luvisol	Fs	30_50
HEF046_17	4.19	46.55	0	46.55	3.63	12.81	387	570	45	1.0506	Luvisol	Fs	30_50
HEF047_11	4.79	32.51	0	32.51	2.43	13.35	323	632	46	0.8971	Stagnosol	Fs	50_100
HEF047_17	4.86	33.95	0	33.95	2.57	13.22	323	632	46	0.8971	Stagnosol	Fs	50_100
HEF048_11	4.13	26.65	0	26.65	2.02	13.21	273	687	44	0.8371	Stagnosol	Fs	50_100
HEF048_17	4.42	37.01	0	37.01	2.47	14.97	273	687	44	0.8371	Stagnosol	Fs	50_100
HEF049_11	3.89	23.82	0	23.82	1.61	14.83	223	732	48	1.2523	Stagnosol	Fs	50_100
HEF049_17	4.07	30.28	0	30.28	2.02	14.99	223	732	48	1.2523	Stagnosol	Fs	50_100
HEF050_11	4.75	33.56	0.23	33.32	2.8	11.91	349	606	46	0.4231	Stagnosol	Fs	50_100
HEF050_17	4.77	42.94	0	42.94	3.57	12.03	349	606	46	0.4231	Stagnosol	Fs	50_100
SEF001_11	3.5	18.6	0	18.6	0.8	23.2	37	81	882	1.9003	Cambisol	Ps	15_30
SEF001_17	3.64	23.28	0	23.28	1.05	22.18	37	81	882	1.9003	Cambisol	Ps	15_30
SEF002_11	3.4	20.56	0	20.56	1.2	17.07	32	98	870	1.4052	Cambisol	Ps	30_50
SEF002_17	3.52	25.01	0	25.01	1.47	16.98	32	98	870	1.4052	Cambisol	Ps	30_50
SEF003_11	3.34	20.54	0	20.54	1.03	19.84	24	50	926	1.8981	Cambisol	Ps	30_50
SEF003_17	3.44	21.41	0	21.41	1.03	20.76	24	50	926	1.8981	Cambisol	Ps	30_50
SEF004_11	3.27	24.63	0	24.63	1.19	20.75	27	54	919	1.5961	Cambisol	Ps	50_100

SEF004_17	3.46	24.81	0	24.81	1.24	19.94	27	54	919	1.5961	Cambisol	Ps	50_100
SEF005_11	3.18	25.04	0	25.04	1.29	19.37	19	17	964	0.689	Cambisol	Fs	50_100
SEF005_17	3.36	25.4	0	25.4	1.32	19.23	19	17	964	0.689	Cambisol	Fs	50_100
SEF006_11	3.45	25.87	0	25.87	1.49	17.39	49	77	874	1.4018	Cambisol	Fs	50_100
SEF006_17	3.66	27.93	0	27.93	1.68	16.59	49	77	874	1.4018	Cambisol	Fs	50_100
SEF007_11	3.49	18.65	0	18.65	1.15	16.29	16	124	860	0.0855	Cambisol	Fs	50_100
SEF007_17	3.75	24.22	0	24.22	1.55	15.65	16	124	860	0.0855	Cambisol	Fs	50_100
SEF008_11	3.28	27.09	0	27.09	1.64	16.54	47	198	755	0.1873	Albeluvisol	Fs	50_100
SEF008_17	3.37	24.19	0	24.19	1.5	16.15	47	198	755	0.1873	Albeluvisol	Fs	50_100
SEF009_11	3.27	20.01	0	20.01	1.04	19.18	0	58	942	0.6459	Cambisol	Fs	50_100
SEF009_17	3.49	16.87	0	16.87	0.91	18.57	0	58	942	0.6459	Cambisol	Fs	50_100
SEF010_11	3.58	18.31	0	18.31	1.02	17.94	36	53	911	1.1101	Cambisol	Ps	15_30
SEF010_17	3.72	23.46	0	23.46	1.23	19.07	36	53	911	1.1101	Cambisol	Ps	15_30
SEF011_11	3.29	31.32	0	31.32	1.31	23.83	31	85	884	2.1194	Cambisol	Ps	15_30
SEF011_17	3.67	16.08	0	16.08	0.77	20.99	31	85	884	2.1194	Cambisol	Ps	15_30
SEF012_11	3.38	20.76	0	20.76	0.79	26.3	18	106	876	2.2966	Cambisol	Ps	15_30
SEF012_17	3.5	22.62	0	22.62	0.9	25.24	18	106	876	2.2966	Cambisol	Ps	15_30
SEF013_11	3.16	26.57	0	26.57	1.29	20.53	28	51	921	1.1203	Podzol	Ps	30_50
SEF013_17	3.25	26.44	0	26.44	1.25	21.15	28	51	921	1.1203	Podzol	Ps	30_50
SEF014_11	3.18	25.28	0	25.28	1.43	17.63	8	17	975	1.7147	Cambisol	Ps	30_50
SEF014_17	3.38	24.38	0	24.38	1.41	17.3	8	17	975	1.7147	Cambisol	Ps	30_50
SEF015_11	3.46	10.68	0	10.68	0.55	19.57	31	46	923	1.7954	Cambisol	Ps	30_50
SEF015_17	3.68	10.82	0	10.82	0.63	17.14	31	46	923	1.7954	Cambisol	Ps	30_50
SEF016_11	3.41	19.44	0	19.44	1.06	18.28	30	108	862	1.1101	Cambisol	Ps	50_100
SEF016_17	3.6	14.97	0	14.97	0.84	17.75	30	108	862	1.1101	Cambisol	Ps	50_100
SEF017_11	3.08	21.75	0	21.75	0.92	23.57	24	49	927	1.4928	Cambisol	Ps	50_100
SEF017_17	3.34	20.39	0	20.39	0.9	22.6	24	49	927	1.4928	Cambisol	Ps	50_100
SEF018_11	3.42	31.35	0	31.35	1.43	21.97	29	34	941	1.7025	Cambisol	Ps	30_50
SEF018_17	3.31	27.44	0	27.44	1.28	21.38	29	34	941	1.7025	Cambisol	Ps	30_50
SEF019_11	3.51	6.34	0	6.34	0.31	20.17	37	44	919	2.1162	Cambisol	Ps	30_50
SEF019_17	3.64	15.63	0	15.63	0.75	20.94	37	44	919	2.1162	Cambisol	Ps	30_50
SEF020_11	3.59	16.34	0	16.34	0.69	23.59	42	70	892	1.8772	Regosol	Ps	30_50
SEF020_17	3.64	20.65	0	20.65	0.93	22.21	42	70	892	1.8772	Regosol	Ps	30_50
SEF021_11	3.15	25.43	0	25.43	1.37	18.6	55	69	876	1.9226	Cambisol	Ps	50_100
SEF021_17	3.29	23.49	0	23.49	1.23	19.06	55	69	876	1.9226	Cambisol	Ps	50_100
SEF022_11	3.37	23.37	0	23.37	1.31	17.86	34	71	895	0.7747	Cambisol	Qs	30_50
SEF022_17	3.56	24.94	0	24.94	1.42	17.54	34	71	895	0.7747	Cambisol	Qs	30_50
SEF023_11	3.24	19.62	0	19.62	1.3	15.1	51	76	873	0.9618	Cambisol	Qs	50_100
SEF023_17	3.48	18.42	0	18.42	1.28	14.44	51	76	873	0.9618	Cambisol	Qs	50_100
SEF024_11	3.44	18.17	0	18.17	1.15	15.8	46	142	812	0.6409	Cambisol	Qs	50_100
SEF024_17	3.66	10.48	0	10.48	0.68	15.31	46	142	812	0.6409	Cambisol	Qs	50_100
SEF025_11	3.44	20.69	0	20.69	1.32	15.72	63	77	860	0.853	Cambisol	Qs	30_50
SEF025_17	3.72	21.85	0	21.85	1.37	15.93	63	77	860	0.853	Cambisol	Qs	30_50
SEF026_11	3.52	17.62	0	17.62	1.16	15.18	52	66	886	0.376	Cambisol	Qs	50_100
SEF026_17	3.67	20	0	20	1.33	15.01	52	66	886	0.376	Cambisol	Qs	50_100
SEF027_11	3.3	18.2	0	18.2	1.14	15.93	51	139	810	1.1539	Cambisol	Qs	50_100
SEF027_17	3.43	17.8	0	17.8	1.11	15.97	51	139	810	1.1539	Cambisol	Qs	50_100
SEF028_11	3.36	24.27	0	24.27	1.64	14.76	51	141	808	1.0689	Cambisol	Qs	50_100
SEF028_17	3.52	18.04	0	18.04	1.25	14.38	51	141	808	1.0689	Cambisol	Qs	50_100
SEF029_11	3.18	12.02	0	12.02	0.55	21.93	30	11	959	1.547	Cambisol	Ps	50_100
SEF029_17	3.32	20.44	0	20.44	0.87	23.63	30	11	959	1.547	Cambisol	Ps	50_100
SEF030_11	3.12	26.81	0	26.81	1.16	23.06	50	172	778	1.3926	Cambisol	Ps	50_100
SEF030_17	3.44	19.29	0	19.29	0.94	20.58	50	172	778	1.3926	Cambisol	Ps	50_100
SEF031_11	3.23	20.53	0	20.53	1.06	19.37	51	61	888	1.8482	Cambisol	Ps	50_100

SEF031_17	3.41	28.22	0	28.22	1.41	20.03	51	61	888	1.8482	Cambisol	Ps	50_100
SEF032_11	3.21	28.55	0	28.55	1.44	19.78	51	141	808	1.7734	Cambisol	Ps	50_100
SEF032_17	3.53	22.63	0	22.63	1.14	19.92	51	141	808	1.7734	Cambisol	Ps	50_100
SEF033_11	3.18	12.55	0	12.55	0.5	24.96	25	9	966	1.7008	Cambisol	Ps	50_100
SEF033_17	3.35	11.64	0	11.64	0.48	24.18	25	9	966	1.7008	Cambisol	Ps	50_100
SEF034_11	3.22	16.44	0	16.44	0.74	22.22	42	69	889	1.7943	Albeluvisol	Ps	50_100
SEF034_17	3.46	16.91	0	16.91	0.75	22.57	42	69	889	1.7943	Albeluvisol	Ps	50_100
SEF035_11	3.43	20.64	0	20.64	1.1	18.82	65	74	861	1.0252	Cambisol	Fs	50_100
SEF035_17	3.64	19.11	0	19.11	1.04	18.29	65	74	861	1.0252	Cambisol	Fs	50_100
SEF036_11	3.21	30.36	0	30.36	1.76	17.26	64	88	848	1.2034	Cambisol	Fs	30_50
SEF036_17	3.29	30.93	0	30.93	1.77	17.51	64	88	848	1.2034	Cambisol	Fs	30_50
SEF037_11	3.43	21.34	0	21.34	1.26	16.97	63	70	871	0.6602	Cambisol	Fs	50_100
SEF037_17	3.56	22.95	0	22.95	1.27	18.08	63	70	871	0.6602	Cambisol	Fs	50_100
SEF038_11	3.18	21.87	0	21.87	1.37	15.96	57	103	840	0.772	Cambisol	Fs	50_100
SEF038_17	3.37	22.81	0	22.81	1.4	16.24	57	103	840	0.772	Cambisol	Fs	50_100
SEF039_11	3.46	18.94	0	18.94	1.07	17.74	52	98	850	1.0994	Cambisol	Fs	50_100
SEF039_17	3.73	19.19	0	19.19	1.18	16.33	52	98	850	1.0994	Cambisol	Fs	50_100
SEF040_11	3.56	18.97	0	18.97	1.07	17.73	63	71	866	1.3649	Cambisol	Fs	50_100
SEF040_17	3.77	21.49	0	21.49	1.26	17.03	63	71	866	1.3649	Cambisol	Fs	50_100
SEF041_11	3.57	17.59	0	17.59	0.93	18.96	50	49	905	1.1019	Cambisol	Fs	50_100
SEF041_17	3.78	21.47	0	21.47	1.22	17.54	50	49	905	1.1019	Cambisol	Fs	50_100
SEF042_11	3.55	23.2	0	23.2	1.38	16.85	74	247	679	0.7863	Regosol	Fs	50_100
SEF042_17	3.75	24.71	0	24.71	1.5	16.48	74	247	679	0.7863	Regosol	Fs	50_100
SEF043_11	3.48	18.41	0	18.41	1.12	16.49	69	121	810	1.0317	Cambisol	Fs	50_100
SEF043_17	3.67	16.96	0	16.96	1.04	16.31	69	121	810	1.0317	Cambisol	Fs	50_100
SEF044_11	3.52	17.32	0	17.32	0.94	18.47	75	69	856	0.7971	Cambisol	Fs	50_100
SEF044_17	3.74	23.31	0	23.31	1.34	17.34	75	69	856	0.7971	Cambisol	Fs	50_100
SEF045_11	3.5	16.51	0	16.51	0.92	17.9	48	75	881	0.3413	Albeluvisol	Fs	50_100
SEF045_17	3.71	17.17	0	17.17	1.08	15.92	48	75	881	0.3413	Albeluvisol	Fs	50_100
SEF046_11	3.3	21.2	0	21.2	1.34	15.87	63	132	805	0.2069	Albeluvisol	Fs	50_100
SEF046_17	3.49	24.08	0	24.08	1.48	16.27	63	132	805	0.2069	Albeluvisol	Fs	50_100
SEF047_11	3.21	23.5	0	23.5	1.33	17.62	49	18	933	0.0156	Cambisol	Fs	50_100
SEF047_17	3.45	20.56	0	20.56	1.17	17.63	49	18	933	0.0156	Cambisol	Fs	50_100
SEF048_11	3.5	19.33	0	19.33	1.17	16.46	85	190	725	0.4675	Cambisol	Fs	50_100
SEF048_17	3.67	23.36	0	23.36	1.26	18.59	85	190	725	0.4675	Cambisol	Fs	50_100
SEF049_11	3.24	21.39	0	21.39	1.15	18.52	68	74	858	1.0371	Cambisol	Fs	50_100
SEF049_17	3.47	26.53	0	26.53	1.44	18.42	68	74	858	1.0371	Cambisol	Fs	50_100
SEF050_11	3.41	16.73	0	16.73	1.04	16.08	79	99	822	1.1073	Cambisol	Fs	50_100
SEF050_17	3.68	21.17	0	21.17	1.34	15.85	79	99	822	1.1073	Cambisol	Fs	50_100

1: in sites SEF010 and SEF016 (2011 and 2017) , missing values were replaced by the median (1.1101)

2: Diameter in cm of the trunks of 100 best developed tress of the dominant species
