# Supplementary material for: Contrasting Responses of Protistan Plant Parasites and Phagotrophs to Ecosystems, Land Management and Soil Properties
Source: Front Microbiol. 2020 Aug 5;11:1823. doi: 10.3389/fmicb.2020.01823 (PMC7422690; doi:10.3389/fmicb.2020.01823)
Supplement: Supplementary file 3 [file Data_Sheet_3.zip › Table S3.pdf]

**Table S3**. Average and standard deviation of the continuous edaphic and environmental variables from Tables S1 & S2, by ecosystem (150 sites each), region (50 sites each) and year of sampling. Significant differences between regions are indicated by a different letter. Regional differences are important, while there is little variation between years.

| Ecosystem | Year | Region | pH | Organic Carbon | C/N ratio | Clay | LUI | Fertilization | Mowing |
|---|---|---|---|---|---|---|---|---|---|
| **Grassland** | **2011** | **All** | **6.51 ± 0.73** | **72.19 ± 64.17** | **10.45 ± 0.88** | **377.49 ± 192.39** | **1.61 ± 0.63** | **35.48 ± 53.59** | **1.03 ± 0.94** |
| | | Alb | 6.27 ± 0.55 a | 65.13 ± 13.90 b | 10.30 ± 0.76 a | 536.06 ± 134.02 c | 1.62 ± 0.61 a | 48.75 ± 61.51 b | 1.20 ± 0.99 a |
| | | Hainich | 6.89 ± 0.51 b | 45.62 ± 12.13 a | 10.26 ± 0.54 a | 422.58 ± 130.26 b | 1.60 ± 0.67 a | 46.99 ± 59.00 b | 0.94 ± 0.91 a |
| | | Schorfheide | 6.39 ± 0.91 a | 105.83 ± 101.27 c | 10.80 ± 1.13 b | 173.84 ± 86.98 a | 1.62 ± 0.63 a | 10.70 ± 22.97 a | 0.94 ± 0.91 a |
| | **2017** | **All** | **6.59 ± 0.74** | **71.69 ± 56.56** | **9.99 ± 1.04** | **same as 2011** | **3.00 ± 3.48** | **23.32 ± 57.29** | **1.08 ± 0.92** |
| | | Alb | 6.33 ± 0.60 a | 69.60 ± 14.25 b | 10.38 ± 0.71 b | | 3.00 ± 2.56 a | 38.55 ± 82.77 b | 1.30 ± 1.07 b |
| | | Hainich | 6.92 ± 0.48 b | 49.90 ± 12.07 a | 10.23 ± 0.54 b | | 3.00 ± 2.14 a | 30.97 ± 47.91 b | 1.14 ± 0.86 ab |
| | | Schorfheide | 6.52 ± 0.93 a | 95.56 ± 91.15 b | 9.37 ± 1.36 a | | 3.00 ± 5.08 a | 0.44 ± 2.19 a | 0.80 ± 0.73 a |
| | | | | | | | | | |
| **Forest** | **2011** | **All** | **4.49 ± 1.07** | **39.51 ± 20.11** | **15.03 ± 3.23** | **280.66 ± 203.34** | | | |
| | | Alb | 5.30 ± 0.76 c | 62.06 ± 14.28 c | 13.04 ± 0.92 a | 496.06 ± 104.83 c | | | |
| | | Hainich | 4.81 ± 0.88 b | 35.65 ± 10.49 b | 13.24 ± 1.07 a | 301.10 ± 99.27 b | | | |
| | | Schorfheide | 3.36 ± 0.14 a | 20.81 ± 5.01 a | 18.82 ± 2.80 b | 44.82 ± 18.86 a | | | |
| | **2017** | **All** | **4.67 ± 1.08** | **42.53 ± 20.13** | **15.12 ± 2.96** | **same as 2011** | | | |
| | | Alb | 5.49 ± 0.81 c | 62.42 ± 13.46 c | 13.22 ± 0.88 a | | | | |
| | | Hainich | 4.98 ± 0.88 b | 43.84 ± 12.94 b | 13.64 ± 1.14 a | | | | |
| | | Schorfheide | 3.54 ± 0.15 a | 21.32 ± 4.41 a | 18.48 ± 2.66 b | | | | |
