# Supplementary material for: Contrasting Responses of Protistan Plant Parasites and Phagotrophs to Ecosystems, Land Management and Soil Properties
Source: Front Microbiol. 2020 Aug 5;11:1823. doi: 10.3389/fmicb.2020.01823 (PMC7422690; doi:10.3389/fmicb.2020.01823)
Supplement: Supplementary file 3 [file Data_Sheet_3.zip › Table S4.pdf]

Table S4. Primers and combinantion of barcodes used to identify soil samples. A stands for Schwäbische Alb; H for Hainich and S for Schorfheide-Chorin. G stands for grassland, F for forest.

Primer	GTTAAAARGCTCGTAGTYG	AAGARGAYATCCTTGGTG	Grassland sites	Forest sites
barcode	GTGAACTC	GTCAGTAT	AEG001	AEF001
barcode	GTGAACTC	TACGCTAT	AEG002	AEF002
barcode	GTGAACTC	TTAGGAAC	AEG003	AEF003
barcode	GTGAACTC	GTAACATG	AEG004	AEF004
barcode	GTGAACTC	GACATATC	AEG005	AEF005
barcode	GTGAACTC	TACTGTAG	AEG006	AEF006
barcode	GTGAACTC	TCTCTCAG	AEG007	AEF007
barcode	GTGAACTC	ACGATCAG	AEG008	AEF008
barcode	GCGTAATC	TATCAGTC	AEG009	AEF009
barcode	GCGTAATC	GCTTCAAT	AEG010	AEF010
barcode	GCGTAATC	AATCAGGT	AEG011	AEF011
barcode	GCGTAATC	ACAATGTG	AEG012	AEF012
barcode	GCGTAATC	AATTGGTC	AEG013	AEF013
barcode	GCGTAATC	AAGCTACT	AEG014	AEF014
barcode	GCGTAATC	ATTCTCGG	AEG015	AEF015
barcode	GCGTAATC	GTGTCAAC	AEG016	AEF016
barcode	ATGTGACC	TACGCTAT	AEG017	AEF017
barcode	ATGTGACC	TTAGGAAC	AEG018	AEF018
barcode	ATGTGACC	GTAACATG	AEG019	AEF019
barcode	ATGTGACC	GACATATC	AEG020	AEF020
barcode	ATGTGACC	TACTGTAG	AEG021	AEF021
barcode	ATGTGACC	TCTCTCAG	AEG022	AEF022
barcode	ATGTGACC	CGTTCAAG	AEG023	AEF023
barcode	ATGTGACC	ACGATCAG	AEG024	AEF024
barcode	ATACGCAC	TATCAGTC	AEG025	AEF025
barcode	ATACGCAC	GCTTCAAT	AEG026	AEF026
barcode	ATACGCAC	AATCAGGT	AEG027	AEF027
barcode	ATACGCAC	CCATTATG	AEG028	AEF028
barcode	ATACGCAC	ACAATGTG	AEG029	AEF029
barcode	ATACGCAC	AATTGGTC	AEG030	AEF030
barcode	ATACGCAC	ATTCTCGG	AEG031	AEF031
barcode	ATACGCAC	GTGTCAAC	AEG032	AEF032
barcode	ATCTGGAC	TACGCTAT	AEG033	AEF033
barcode	ATCTGGAC	TTAGGAAC	AEG034	AEF034
barcode	ATCTGGAC	GTAACATG	AEG035	AEF035
barcode	ATCTGGAC	GACATATC	AEG036	AEF036
barcode	ATCTGGAC	TACTGTAG	AEG037	AEF037
barcode	ATCTGGAC	TCTCTCAG	AEG038	AEF038
barcode	ATCTGGAC	CGTTCAAG	AEG039	AEF039
barcode	ATCTGGAC	ACGATCAG	AEG040	AEF040
barcode	GTCAATCC	GCTTCAAT	AEG041	AEF041
barcode	GTCAATCC	AATCAGGT	AEG042	AEF042
barcode	GTCAATCC	CCATTATG	AEG043	AEF043
barcode	GTCAATCC	ACAATGTG	AEG044	AEF044

barcode	GTCAATCC	AATTGGTC	AEG045	AEF045
barcode	GTCAATCC	AAGCTACT	AEG046	AEF046
barcode	GTCAATCC	ATTCTCGG	AEG047	AEF047
barcode	GTCAATCC	GTGTCAAC	AEG048	AEF048
barcode	GTCAATCC	TATCAGTC	AEG049	AEF049
barcode	ATGTGACC	GTCAGTAT	AEG050	AEF050
barcode	GACCATAC	TACGCTAT	HEG001	HEF001
barcode	GACCATAC	TTAGGAAC	HEG002	HEF002
barcode	GACCATAC	GTAACATG	HEG003	HEF003
barcode	GACCATAC	GACATATC	HEG004	HEF004
barcode	GACCATAC	TACTGTAG	HEG005	HEF005
barcode	GACCATAC	TCTCTCAG	HEG006	HEF006
barcode	GACCATAC	CGTTCAAG	HEG007	HEF007
barcode	GACCATAC	ACGATCAG	HEG008	HEF008
barcode	GAATACTC	TATCAGTC	HEG009	HEF009
barcode	GAATACTC	GCTTCAAT	HEG010	HEF010
barcode	GAATACTC	AATCAGGT	HEG011	HEF011
barcode	GAATACTC	CCATTATG	HEG012	HEF012
barcode	GAATACTC	ACAATGTG	HEG013	HEF013
barcode	GAATACTC	AAGCTACT	HEG014	HEF014
barcode	GAATACTC	ATTCTCGG	HEG015	HEF015
barcode	GAATACTC	GTGTCAAC	HEG016	HEF016
barcode	GTTATAGC	GTCAGTAT	HEG017	HEF017
barcode	GTTATAGC	TTAGGAAC	HEG018	HEF018
barcode	GTTATAGC	GTAACATG	HEG019	HEF019
barcode	GTTATAGC	GACATATC	HEG020	HEF020
barcode	GTTATAGC	TACTGTAG	HEG021	HEF021
barcode	GTTATAGC	TCTCTCAG	HEG022	HEF022
barcode	GTTATAGC	CGTTCAAG	HEG023	HEF023
barcode	GTTATAGC	ACGATCAG	HEG024	HEF024
barcode	TAGTTACC	TATCAGTC	HEG025	HEF025
barcode	TAGTTACC	GCTTCAAT	HEG026	HEF026
barcode	TAGTTACC	CCATTATG	HEG027	HEF027
barcode	TAGTTACC	ACAATGTG	HEG028	HEF028
barcode	TAGTTACC	AATTGGTC	HEG029	HEF029
barcode	TAGTTACC	AAGCTACT	HEG030	HEF030
barcode	TAGTTACC	ATTCTCGG	HEG031	HEF031
barcode	TAGTTACC	GTGTCAAC	HEG032	HEF032
barcode	AACTTAGC	TACGCTAT	HEG033	HEF033
barcode	AACTTAGC	TTAGGAAC	HEG034	HEF034
barcode	AACTTAGC	GTAACATG	HEG035	HEF035
barcode	AACTTAGC	GACATATC	HEG036	HEF036
barcode	AACTTAGC	TACTGTAG	HEG037	HEF037
barcode	AACTTAGC	TCTCTCAG	HEG038	HEF038
barcode	AACTTAGC	CGTTCAAG	HEG039	HEF039
barcode	AACTTAGC	ACGATCAG	HEG040	HEF040
barcode	TATCTAGC	GCTTCAAT	HEG041	HEF041
barcode	TATCTAGC	AATCAGGT	HEG042	HEF042
barcode	TATCTAGC	CCATTATG	HEG043	HEF043

barcode	TATCTAGC	ACAATGTG	HEG044	HEF044
barcode	TATCTAGC	AATTGGTC	HEG045	HEF045
barcode	TATCTAGC	AAGCTACT	HEG046	HEF046
barcode	TATCTAGC	ATTCTCGG	HEG047	HEF047
barcode	TATCTAGC	GTGTCAAC	HEG048	HEF048
barcode	AACTTAGC	GTCAGTAT	HEG049	HEF049
barcode	GACCATAC	GTCAGTAT	HEG050	HEF050
barcode	TGATACAC	GTCAGTAT	SEG001	SEF001
barcode	TGATACAC	TACGCTAT	SEG002	SEF002
barcode	TGATACAC	TTAGGAAC	SEG003	SEF003
barcode	TGATACAC	GTAACATG	SEG004	SEF004
barcode	TGATACAC	GACATATC	SEG005	SEF005
barcode	TGATACAC	TACTGTAG	SEG006	SEF006
barcode	TGATACAC	TCTCTCAG	SEG007	SEF007
barcode	TGATACAC	CGTTCAAG	SEG008	SEF008
barcode	GTAGATAC	GCTTCAAT	SEG009	SEF009
barcode	GTAGATAC	AATCAGGT	SEG010	SEF010
barcode	GTAGATAC	CCATTATG	SEG011	SEF011
barcode	GTAGATAC	ACAATGTG	SEG012	SEF012
barcode	GTAGATAC	AATTGGTC	SEG013	SEF013
barcode	GTAGATAC	AAGCTACT	SEG014	SEF014
barcode	GTAGATAC	ATTCTCGG	SEG015	SEF015
barcode	GTAGATAC	GTGTCAAC	SEG016	SEF016
barcode	AGTTCATC	GTCAGTAT	SEG017	SEF017
barcode	AGTTCATC	TACGCTAT	SEG018	SEF018
barcode	AGTTCATC	TTAGGAAC	SEG019	SEF019
barcode	AGTTCATC	GTAACATG	SEG020	SEF020
barcode	AGTTCATC	GACATATC	SEG021	SEF021
barcode	AGTTCATC	TACTGTAG	SEG022	SEF022
barcode	AGTTCATC	CGTTCAAG	SEG023	SEF023
barcode	AGTTCATC	ACGATCAG	SEG024	SEF024
barcode	TTAGAGTC	GCTTCAAT	SEG025	SEF025
barcode	TTAGAGTC	AATCAGGT	SEG026	SEF026
barcode	TTAGAGTC	CCATTATG	SEG027	SEF027
barcode	TTAGAGTC	ACAATGTG	SEG028	SEF028
barcode	TTAGAGTC	AATTGGTC	SEG029	SEF029
barcode	TTAGAGTC	AAGCTACT	SEG030	SEF030
barcode	TTAGAGTC	ATTCTCGG	SEG031	SEF031
barcode	TTAGAGTC	GTGTCAAC	SEG032	SEF032
barcode	TATATGCC	TACGCTAT	SEG033	SEF033
barcode	TATATGCC	TTAGGAAC	SEG034	SEF034
barcode	TATATGCC	GTAACATG	SEG035	SEF035
barcode	TATATGCC	GACATATC	SEG036	SEF036
barcode	TATATGCC	TACTGTAG	SEG037	SEF037
barcode	TATATGCC	TCTCTCAG	SEG038	SEF038
barcode	TATATGCC	CGTTCAAG	SEG039	SEF039
barcode	TATATGCC	ACGATCAG	SEG040	SEF040
barcode	AATTGGTC	TATCAGTC	SEG041	SEF041
barcode	AATTGGTC	GCTTCAAT	SEG042	SEF042

barcode	AATTGGTC	AATCAGGT	SEG043	SEF043
barcode	AATTGGTC	CCATTATG	SEG044	SEF044
barcode	AATTGGTC	ACAATGTG	SEG045	SEF045
barcode	AATTGGTC	AATTGGTC	SEG046	SEF046
barcode	AATTGGTC	AAGCTACT	SEG047	SEF047
barcode	AATTGGTC	ATTCTCGG	SEG048	SEF048
barcode	TATATGCC	GTCAGTAT	SEG049	SEF049
barcode	TTAGAGTC	TATCAGTC	SEG050	SEF050
barcode	GTAGATAC	TATCAGTC	MOCK	MOCK
