# Supplementary material for: Contrasting Responses of Protistan Plant Parasites and Phagotrophs to Ecosystems, Land Management and Soil Properties
Source: Front Microbiol. 2020 Aug 5;11:1823. doi: 10.3389/fmicb.2020.01823 (PMC7422690; doi:10.3389/fmicb.2020.01823)
Supplement: Supplementary file 3 [file Data_Sheet_3.zip › Table S6.pdf]

Table S6. Most parsimonious models (dbRDA), with their respective R^2 adjusted and F values, and the F values of the factors selected by each model. Significance values shown as symbol (see footnote).

CERCOZOA & ENDOMYXA

Grassland	R^2 (%)	Anova F value	soil type	pH	clay	manage-ment	LUI	fertili-zation	mowing	C/N ratio
**All OTUs**	5.5	2.4 ***	2.3 **			1.6 *	2.0 **	2.0 **		3.5 **
**Bacterivores**	4.2	2.6 ***	2.6 *							2.9 *
**Omnivores**	5.2	2.6 ***	2.6 **			1.7 *				4.3 **
**Eukaryvores**	6.0	2.8 ***	2.7 **			2.5 **				4.9 **
**Plant parasites**	6.3	3.2 ***	3.2 **			2.5 **				

Grassland by region	R^2 (%)	Anova F value	soil type	pH	clay	manage-ment	LUI	fertili-zation	mowing	C/N ratio
**Alb**	NS	NS								
**Hainich**	7.3	3.0 ***	3.6 ***				2.9 **		4.0 ***	
**Schorfheide**	3.6	1.6 **	1.6 **		2.1 **		1.9 *			2.6 **

Forest	R^2 (%)	Anova F value	soil type	pH	clay	main tree species	intensity manag.	develop-mental stage	organic C	C/N ratio
**All OTUs**	47.7	23.1 ***	3.8 ***	17.1 ***	9.3 ***	5.8 ***				6.0 **
**Bacterivores**	44.4	18.9 ***	3.7 **	10.8 **	7.2 **	5.5 **			2.6 *	5.5 **
**Omnivores**	53.2	28.7 ***	3.5 **	24.9 **	8.9 **	6.3 **				6.2 **
**Eukaryvores**	42.6	19.0 ***	4.1 **	8.1 **	8.2 **	4.9 **				4.9 **

Forest by region	R^2 (%)	Anova F value	soil type	pH	clay	main tree species	intensity manag.	develop-mental stage	organic C	C/N ratio
**Alb**	25.9	7.9 ***		19.9 ***	4.0 **	4.8 ***			2.4 *	8.5 ***
**Hainich**	10.2	4.4 ***		4.6 **	4.0 **	1.9 *			5.3 **	
**Schorfheide**	28.7	4.2 ***	1.5 **	12.9 **	1.8 *	3.6 **		2.1 **	3.2 *	2.7 **

*p values signification codes: *** ≤ 0.001; ** ≤ 0.01; * ≤ 0.05.*
